# The Chromatin Architectural Protein CTCF Is Critical for Cell Survival upon Irradiation-Induced DNA Damage

**DOI:** 10.3390/ijms23073896

**Published:** 2022-03-31

**Authors:** Stefania Mamberti, Maruthi K. Pabba, Alexander Rapp, M. Cristina Cardoso, Michael Scholz

**Affiliations:** 1 Cell Biology and Epigenetics, Department of Biology, Technical University of Darmstadt, 64287 Darmstadt, Germany; ste.mamberti@gmail.com (S.M.); pabba.maruthi123@gmail.com (M.K.P.); rapp@bio.tu-darmstadt.de (A.R.); 2 Biophysics Department, GSI Helmholtzzentrum für Schwerionenforschung GmbH, 64291 Darmstadt, Germany

**Keywords:** cancer, chromatin architecture, chromatin domain, clonogenic survival, CTCF, DNA damage response, DNA repair, ionizing radiation, radiosensitivity, biophysical modeling

## Abstract

CTCF is a nuclear protein initially discovered for its role in enhancer-promoter insulation. It has been shown to play a role in genome architecture and in fact, its DNA binding sites are enriched at the borders of chromatin domains. Recently, we showed that depletion of CTCF impairs the DNA damage response to ionizing radiation. To investigate the relationship between chromatin domains and DNA damage repair, we present here clonogenic survival assays in different cell lines upon CTCF knockdown and ionizing irradiation. The application of a wide range of ionizing irradiation doses (0–10 Gy) allowed us to investigate the survival response through a biophysical model that accounts for the double-strand breaks’ probability distribution onto chromatin domains. We demonstrate that the radiosensitivity of different cell lines is increased upon lowering the amount of the architectural protein. Our model shows that the deficiency in the DNA repair ability is related to the changes in the size of chromatin domains that occur when different amounts of CTCF are present in the nucleus.

## 1. Introduction

CTCF (CCCTC-binding factor) is an 11-zinc finger protein, which was initially described to bind 3 repeats of the DNA motif CCCTC and thereby to regulate the transcription of the c-myc oncogene [1,2]. The very conserved 11 zinc fingers of CTCF can be combined in different ways to achieve the binding of various sequences, such as the ones of promoters of the same gene, which have diverged during evolution in different species [3]. CTCF was hence initially classified as an insulator protein, due to its ability to insulate proximal DNA sequences (as reviewed in [4,5]). By mediating the insulation of enhancers and promoters, CTCF can either act as a transcriptional activator or repressor [4].

Besides its important role in transcriptional regulation, CTCF has received major interest due to its cooperation with the cohesin complex in chromatin loop formation. According to the loop extrusion model, CTCF acts as a border to the chromatin extruding activity of the ring-shaped cohesin complex, when two CTCF molecules bind two DNA motifs that are convergent in orientation [6].

With the introduction of chromosome conformation capture (3C) and related high-throughput techniques (e.g., Hi-C), matrices of the contact frequencies between different genomic sites have been calculated. Genome-wide interaction maps of CTCF were thus generated under various cellular conditions (reviewed in [7]). It soon became clear that CTCF binding sites are enriched at the boundaries of different types of chromatin domains, ranging from the loop domains [8] through to the topologically associated domains (TADs; [9]) to the A/B compartments [10].

The determinants of such domains have been investigated through depletion of the main architectural proteins and their cofactors and deletion of the genomic loci associated with domain boundaries and subsequent modeling. Since the homozygous knockout of the *CTCF* gene was shown to be lethal [11,12,13], subsequent studies have employed knockdown strategies. Zuin and colleagues proposed that CTCF and cohesin shape TADs in non-redundant ways, with CTCF being necessary for the maintenance of their boundaries and cohesin promoting self-association within the domains but not the segregation of neighboring domains [14]. In different cohesin depletion studies, TADs disappeared [15,16,17] or their borders shifted [18]. Recently, we demonstrated that cohesin depletion does not affect chromosome territories, but the size of DNA loops was enlarged up to 10-fold [19].

Nora and colleagues engineered mouse embryonic stem cells with an auxin-inducible degron to deplete CTCF [20,21] and showed its requirement for those loops with CTCF binding sites at their anchors [21]. CTCF depletion also caused loss of TAD insulation while A/B compartmentalization was mostly unaffected [21]. Additionally, they showed that misfolding defects were dose dependent and that TAD loss required the highest CTCF depletion [21].

The fact that upon depletion of either cohesin or CTCF, TADs are weakened or lost while the A/B compartments are unaffected or even strengthened [14,15,16,17,18,19,21] led the authors of the loop extrusion model to seek an explanation [22]. Through polymer modeling, they concluded that chromatin organization is the result of competition between loop extrusion and phase separation-mediated compartmentalization [22].

CTCF loops were shown to be highly dynamic, based on the very transient and cell cycle-dependent binding time of the protein, as measured by live-cell single-molecule microscopy [23,24]. However, as we reviewed recently [25], this dynamicity does not interfere with the concept of loops as stable structures over time. Their constant act of releasing and reforming will simply give rise to single-cell and cell cycle-dependent variability, which can be overcome by averaging the single-cell snapshots on a population level.

Ionizing radiation is high-energy radiation that is able to directly induce DNA breaks (30–40% of lesions) and generate reactive oxygen species (ROS) that oxidize biological molecules (60–70% of lesions) [26,27,28]. When these free radicals interact with DNA, they can generate abasic sites, base and sugar damage, depolymerization, crosslinking, and single-strand breaks [26,27,28]. Double-strand breaks (DSBs) are considered the most important DNA lesion, as they have the highest probability of inducing cell death, mutation, chromosomal aberrations, and carcinogenic transformation [28,29,30].

Recently, we identified CTCF as a key regulator of the DNA damage response (DDR) upon DSBs [31], which typically starts with a signaling cascade through the phosphorylation of the histone variant H2AX on serine 139 (γH2AX) [32,33]. For each Gray unit of ionizing radiation, corresponding roughly to 30 DSBs, 1% of the total H2AX amount of the genome is phosphorylated, which means that 2 Mbp of chromatin are involved in the response to a single DSB in a G1-phase genome of mammalian cells (0.03% of 6 × 10^9^ bp; [32,33]). H2AX phosphorylation is deposited by the key repair kinases ataxia telangiectasia mutated (ATM), DNA-PKcs, and ATR (ATM and RAD3-related) [34,35] and initiates at DSB sites [36,37] before spreading further [32,38]. ATM, DNA-PKcs, and ATR are correspondingly activated by the DNA-binding complexes MRE11-RAD50-NBS1 (MRN) [39,40], Ku70-Ku86 (XRCC6/XRCC5) [41,42,43], and RPA-ATRIP [44,45], which have respectively high affinity for diverse structures, including DSBs [46], DSB ends [41,43], and extended ssDNA from resected DSBs [44,47]. The γH2AX mark induces the recruitment of the repair factors to the sites of DNA damage, such as 53BP1, BRCA1, and RAD51 [48,49,50,51,52]. The choice between the two main DSB repair pathways of non-homologous end-joining (NHEJ) or homologous recombination (HR) is determined by the cell cycle phase and the chromatin environment [52,53].

Through 3D structured illumination microscopy (3D-SIM), we could resolve the γH2AX foci into clusters of ~4 75-kb-sized nano-foci (median size) [31]. Interestingly, this DNA size corresponds to the one of a single chromatin loop (reviewed in [25]). We found that single γH2AX-labeled loops are clustered together by a couple of CTCF molecules at the anchor of this multi-loop domain [31]. Each cluster of loops contains a single DSB, as shown by the 1:4 ratio of the foci of DSB-binding phospho-Ku70 proteins to γH2AX nano-foci [31]. Additionally, we found that CTCF depletion impairs the spreading of H2AX phosphorylation after damage, decreasing the number of γH2AX nano-foci and their clustering [31].

A different study showed that CTCF supports homologous recombination by associating with BRCA2 in a PARylation-dependent manner, hence enhancing the recruitment of BRCA2 to the sites of DSBs [54]. CTCF accumulated rapidly at DNA lesions following ultraviolet laser micro-irradiation, persisting for up to 2 h [54]. Its depletion delayed the repair kinetics of γH2AX foci but not 53BP1, increased the sensitivity to PARP-1 inhibitors, and decreased the survival potential [54].

In this study, the survival ability of different CTCF-depleted cell lines upon exposure to ionizing radiation was integrated into a biophysical model on the impact of CTCF on cellular radiosensitivity. CTCF levels were quantified prior to and upon CTCF depletion. We compared the survival rates to predictions of a variant of the Giant-LOop-Binary-LEsion model (GLOBLE) [55,56,57], which takes into account the number of giant 2 Mbp domains onto which double-strand breaks have the opportunity to distribute and how the clustering of DNA breaks can affect their repair and lethality. Based on our finding that CTCF holds together the anchors of a multi-loop chromatin domain, in which one single DSB is surrounded by, on average, four γH2AX-decorated chromatin loops [31], the model could explain the increased cell lethality in CTCF-depleted cells upon irradiation well. In fact, the model can predict the observed cell survival data when chromatin domains are supposed to lose coherence upon CTCF depletion and, consequently, multiple DSBs are clustered together, leading to decreased survival.

## 2. Results

### 2.1. Validation of the Genome Size, CTCF Basal Levels, and Binding in Different Cancer Cell Lines

In our previous study based on high-resolution microscopy, we observed that, on average, four loop-sized γH2AX nano-foci cluster around each double-strand break and that the DNA damage response is impaired in cells with diminished amounts of CTCF [31]. We concluded that the ability to signal DNA damage and to repair DSBs relies on CTCF anchoring multi-loop domains [31]. To better understand the relationship between CTCF and radiosensitivity, we aimed to design a model that can predict the clonogenic survival potential when a lower number of domains are anchored by CTCF.

**Figure 1:**
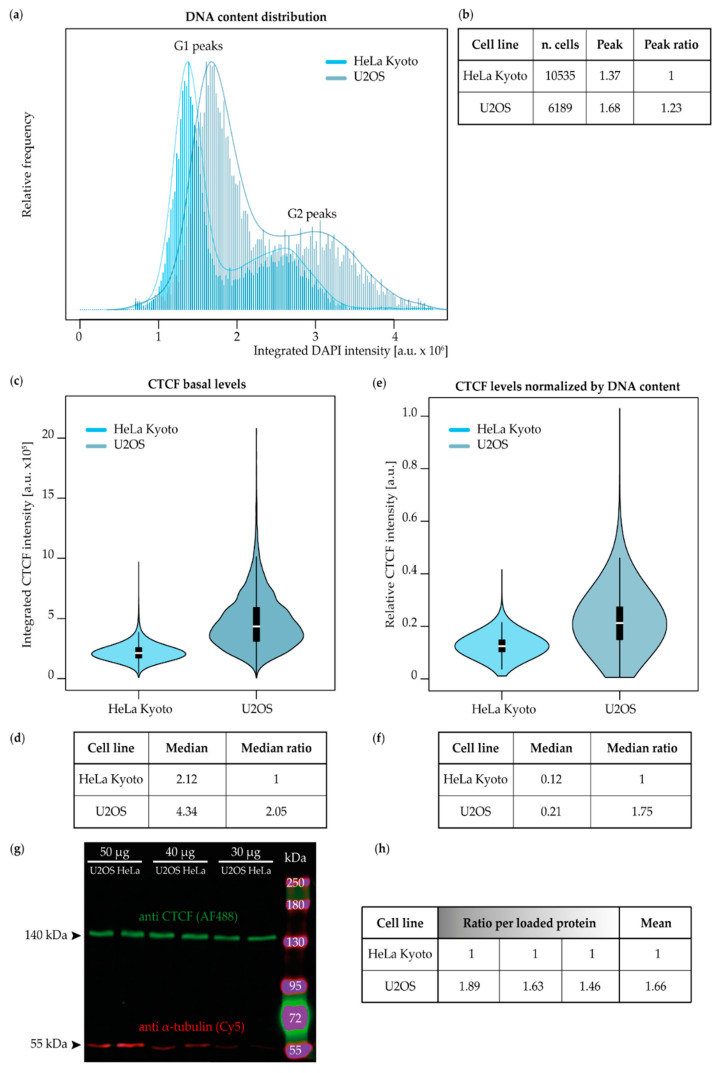
Genome size and CTCF basal levels differ in HeLa Kyoto and U2OS. (**a**–**f**) Untreated cells from the two different cell lines were grown on coverslips, simultaneously fixed, permeabilized, immunostained for CTCF, and DNA counterstained with DAPI (see Table S2 for antibody characteristics). Cells were imaged with high-content wide-field microscopy (see Table S3 for imaging system characteristics) and the total nuclear CTCF and DNA intensities were calculated from the segmented nuclei (see Figure S1 for image analysis pipeline). The number of cells for (**a**–**f**) is shown in (**b**), resulting from two biological replicates (see Table S4 for statistics). As HeLa Kyoto showed the lowest values for both the DNA content and CTCF amounts, this cell line was chosen to calculate the relative ratios for a quantitative comparison. (**a**) DNA content distribution of HeLa Kyoto and U2OS cells showing respective G1 and G2 peaks. The frequency of integrated nuclear DAPI intensities was plotted together with a density function to visualize and compare the genome size distribution of the two cancer cell lines. (**b**) The raw G1 peak measurements of the genome distributions shown in (**a**) and the ratio of the G1 peak of U2OS to the one of HeLa Kyoto are shown. (**c**) Violin plots showing the CTCF basal levels in HeLa Kyoto and U2OS. Total nuclear CTCF intensities were plotted for each cell line, upon subtraction of the background values. The background values were calculated from the intensity levels of cells stained with the primary antibody being omitted. (**d**) Median values relative to (**c**) and the ratio of the U2OS median value to the one of HeLa Kyoto are shown. (**e**) Violin plots showing the total nuclear CTCF intensity upon normalization by the DNA content, i.e., divided by total nuclear DAPI intensities. (**f**) Median values relative to (**e**) and the ratio of the U2OS median value to the one of HeLa Kyoto are shown. (**g**) Quantitative Western blot validating the antibody specificity and confirming the in situ measurements. An SDS-PAGE was run with whole cell lysates of both cell lines loaded in three equal protein amounts. Once blotted on a nitrocellulose membrane, CTCF and α-tubulin bands were detected with fluorescent antibodies (Table S2) and their intensities quantified. (**h**) Each CTCF band value was normalized to the respective α-tubulin one. Normalized U2OS values were divided by the corresponding protein amount in HeLa Kyoto (see Figure S2 for the Western blot analysis). From left to right, the ratios corresponding to 50, 40, and 30 µg of cell lysate loaded are shown. Western blot results consist of one biological replicate (see Figure S2 for the Western blot calculations).

Given the increased necessity of a better understanding of cancer cell dynamics and treatment, we used two different human tumor cell lines: cervical carcinoma (HeLa Kyoto [58]) and osteosarcoma (U2OS [59], Table S1). To measure the impact of CTCF depletion in the two cell lines under study, we first assessed the differences in the genome size and total amount of CTCF. This is particularly important, as tumor cells are known to be genomically unstable. As a DNA sequence-specific binding protein, the amount of CTCF needs to be evaluated relative to the cell’s genome size, which would translate to more or less CTCF binding sites. To this aim, both untreated HeLa Kyoto and U2OS cells were immunostained for CTCF and DNA was counterstained with DAPI (Table S2). Upon high-content microscopy imaging, nuclei were segmented and analyzed for both the DNA content and CTCF basal levels (Figure 1 and Figure S1, Table S3). By plotting the integrated nuclear DNA intensity distribution, we could identify the major G1 and G2 peaks, separated by the population of cells progressing through S-phase. The G1 peak was utilized to calculate the ratio of the higher genome content of U2OS to the lower one of HeLa Kyoto, which yielded a 1.23-fold larger genome size for the U2OS cells (Figure 1a,b). Based on our previous calculations, the genome size of HeLa Kyoto corresponds to 9.7 × 10^3^ Mbp [60] and by multiplying this value by the ratio, we estimated a genome size of 11.93 × 10^3^ Mbp for U2OS.

From the same data set, we extracted the integrated nuclear intensity of CTCF (after background subtraction from samples omitting the primary antibody against CTCF).

We measured a median value of 2.12 and 4.34 a. u. in HeLa Kyoto and U2OS, respectively ( Figure 1c,d and Figure S1, Table S4). Once the median value of U2OS was divided by the one of HeLa Kyoto, we found that the total amount of CTCF in the osteosarcoma cell line is double the one of cervical cancer cells. These findings correlate with the copy number differences (Cancer Cell Line Encyclopedia [61]) and relative gene expression levels (The Human Protein Atlas [62]). We then asked ourselves how this could be impacted by the difference in the genome size and divided the CTCF intensities by the respective integrated DNA values, obtaining a normalized ratio of CTCF of 1.75 in U2OS compared to HeLa Kyoto (Figure 1e,f and Figure S1, Table S4). We validated the specificity of the CTCF antibody through Western blot, detecting a single 140 kDa band in both cell lines (Figure 1g). Moreover, the quantification of the fluorescent CTCF bands from the Western blot resulted in a protein amount that agreed with the immunostaining quantification in situ, with an average U2OS to HeLa Kyoto ratio of 1.66 (Figure 1g,h and Figure S2).

### 2.2. CTCF Knockdown and Validation in Different Cancer Cell Lines

**Figure 2:**
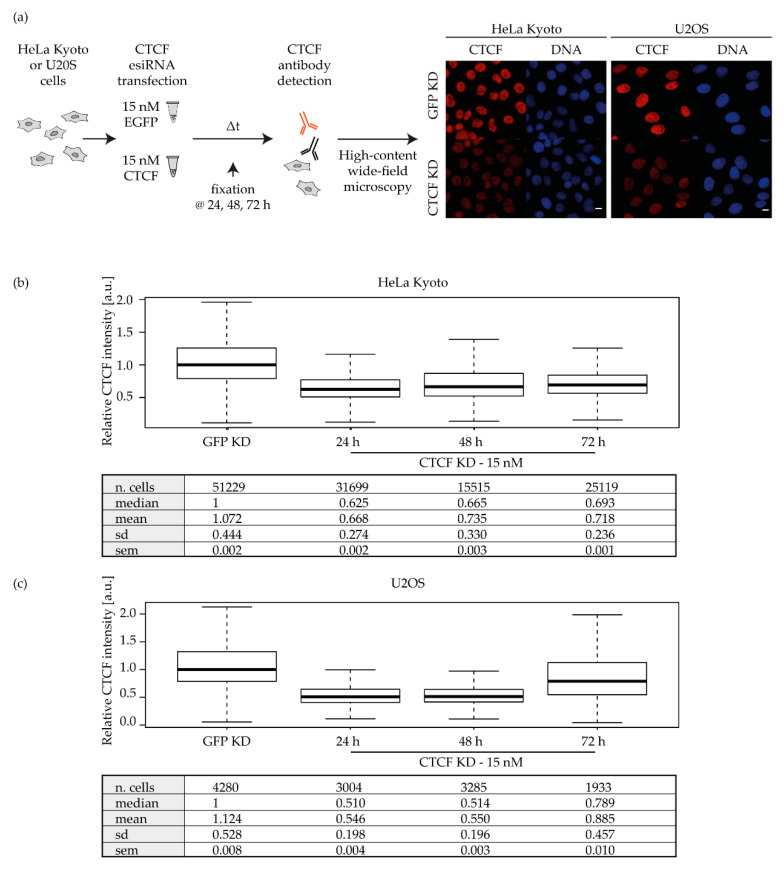
CTCF knockdown is efficiently achieved in 24 h in HeLa Kyoto and U2OS. (**a**) Experimental settings for the knockdown validation. HeLa Kyoto and U2OS cells were transfected with 15 nM of esiRNA against either the human CTCF transcript or EGFP through electroporation and seeded on coverslips. Cells were fixed at 24, 48, or 72 h post transfection and immunostained for CTCF (Table S2). Cells were imaged using a high-content wide-field microscope (scale bar 10 µm; Table S3). (**b**,**c**) Relative CTCF nuclear intensity at different time points upon esiRNA transfection in HeLa Kyoto (**b**) and U2OS (**c**). From the high-content microscopy images, nuclei were masked based on the DAPI signal and the CTCF nuclear intensities were measured (see Figure S1). The integrated sum intensity values were then normalized to the values of the mock-depleted GFP KD sample and plotted as boxplots (sd = standard deviation; sem = standard error of mean); the results consist of three biological replicates (Table S4). See Figure S3 for the boxplot interpretation.

The depletion of CTCF in cancer cells was performed by electroporation-mediated delivery of an esiRNA pool targeting the human CTCF transcript (Figure 2, Table S5). The knockdown was then confirmed by quantitative immunofluorescence staining in situ followed by high-content microscopy analysis (Figure 2a and Figure S1, Tables S2 and S3). In detail, the nuclear sum CTCF values of each sample were normalized (divided) by the median value of the respective EGFP mock-depleted control (hereafter renamed the GFP KD sample).

CTCF depletion was already reached in both HeLa Kyoto and U2OS at 24 h post transfection with 15 nM of esiRNA, with a median depletion of circa 40% and 50% of the control levels, respectively (Figure 2b,c). We could therefore estimate a residual amount of protein corresponding to 60% in HeLa Kyoto and 50% in U2OS (Figure 2b,c, Table S4). At later time points, the same concentration gave similar results (Figure 2b,c), indicating that the depletion was quite stable and that the early time point could be used for the following experiments, i.e., irradiation.

### 2.3. CTCF Depletion Increases the Radiosensitivity of Cancer Cells in a Cell Line-Dependent Way

In our previous studies on the DNA damage response units [31], we observed that CTCF-depleted cells were unable to properly signal the damage through γH2AX, or recruit the repair factors and showed reduced DSB repair upon irradiation. Based on the architectural role of CTCF, we concluded that this deficiency in repairing DNA double-strand breaks was related to the loss of correct chromatin looping.

**Figure 3:**
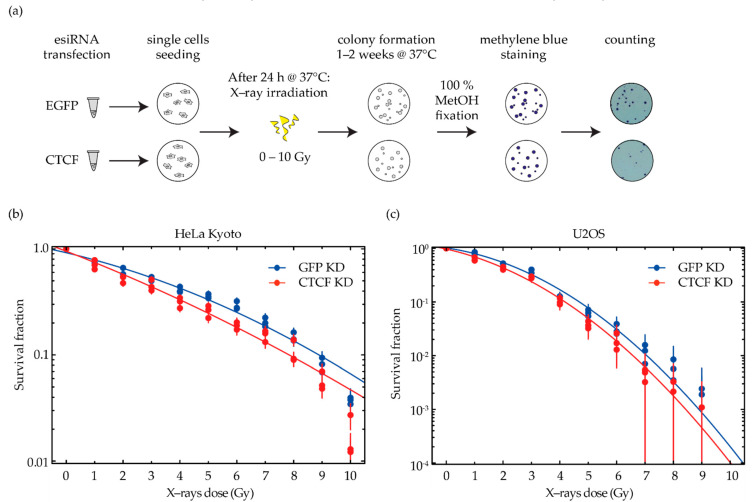
Radiosensitivity is increased, and relative survival is decreased upon CTCF depletion. (**a**) Experimental settings for the clonogenic assay. Cells were transfected with 15 nM of an esiRNA pool against either the human CTCF transcript or against EGFP as a mock treatment (Table S5). From each different transfection, single cells were seeded and allowed to attach before irradiation at the respective time point of maximum depletion for each cell line (40–50% of CTCF depletion at 24 h for HeLa Kyoto and U2OS, Figure 2b,c). Cells were then irradiated with different doses of X-rays and allowed to form colonies for 7–10 days under standard culture conditions. Colonies were then fixed with cold 100% methanol and stained with methylene blue. The colonies with more than 50 cells were microscopically identified and counted by eye. (**b**,**c**) Clonogenic assay results of CTCF-depleted HeLa Kyoto (**b**) and U2OS (**c**). The number of colonies of each technical replicate and treatment was normalized to the average of the respective unirradiated control and plotted as the relative survival fraction in a semi-logarithmic scale. A linear-quadratic dose–response curve fitting weighted by $\frac{1}{relative\;error^{2}}$ was applied. The results are based on three biological replicates, each depicted as a dot in the survival graph and composed of technical replicates (whiskers = error bars, Table S6). Color legend: blue = GFP KD, red = CTCF KD. The same experimental data are plotted with equal y axis scaling in Figure S5a,b for a direct comparison of the 2 cell lines.

Therefore, we decided to further investigate this relationship by testing a range of X-ray doses on CTCF-depleted cancer cells and by elaborating on the survival data to construct a model. In particular, cells were first subjected to 40–50% depletion of the architectural protein CTCF (Figure 2), then exposed to different doses of X-ray irradiation at 24 h post transfection, and allowed to form colonies (Figure 3a).

The survival data of each treatment was normalized to the average of the respective unirradiated control to calculate the survival fraction (see Table S6 for survival data statistics). In general, the survival curves follow the typical linear-quadratic behavior, i.e., $S\left(D\right)=e^{-\left(\alpha D+\beta D^{2}\right)}$, with α representing the initial slope at low doses and β characterizing the bending of the dose–response curve. We found that CTCF-depleted cells of both cell lines had a lower clonogenic potential than mock-treated cells at all radiation doses (Figure 3b,c, Figures S4 and S5a,b, Table S6). Our hypothesis is that the effect of CTCF depletion would be more relevant to the high-dose part of any survival curve, which is shaped by the β term and related to the increased frequency of clustered DSBs. The less CTCF present in the nucleus, holding the borders of chromatin domains, the higher the β term would be. This correlation is hypothesized based on the role of CTCF in shaping chromatin domains and the concomitant distribution of DNA damage within the same or different domain(s). In particular, cervical cancer cells are characterized by a survival curve with a very high α/β ratio (Table 1), meaning that the β term is not dominant in shaping the curve of HeLa Kyoto. We observed a modest increase in the radiosensitivity of this cervical cancer cell line upon CTCF depletion (Figure 3b and Figure S5a, see Table S6 for survival data statistics and Figure S4 for colony formation images).

We then applied the same treatment to the osteosarcoma cell line and expected to observe a higher impact due to the CTCF depletion. U2OS cells are, in fact, known to have a smaller α/β ratio (Table 1) and hence, a more pronounced shoulder in the survival curve compared to HeLa Kyoto (Figure 3c and Figure S5b, Table S6). However, the higher endogenous CTCF levels in U2OS led to a modest difference in survival upon CTCF depletion (Figure 3c and Figure S5b, see Table S6 for survival data statistics and Figure S4 for colony formation images).

### 2.4. GLOBLE Model Investigates the Impact of DSB Clustering in Chromatin Domains on Cell Kill

Our modeling approach is an adaptation of the previously published Giant-LOop-Binary-LEsion (GLOBLE) model (see Section 4.7; [55,56]), which allows quantitative prediction of several aspects of the cellular response to photon radiation. These comprise, e.g., the general linear-quadratic shape of cell survival curves [55,56], the impact of the dose rate on radiosensitivity [57], and the cell cycle dependence of radiosensitivity [64].

The GLOBLE model is based on the premise that the initial clustering properties of primary DNA damage, i.e., DSBs, are predictive of the expected cell killing. Clustering is defined with respect to the multiplicity of DSB within chromatin domains. Based on a given DSB yield and irradiation dose, the multiplicity of DSB within the domains can be determined by Poisson statistics, assuming that the DSBs are randomly distributed within the DNA after photon irradiation. Cases where only single DSBs are found in a domain are called “isolated DSBs” (iDSB) while cases with two or more DSBs in a domain are called “clustered DSBs” (cDSB).

Since CTCF is considered a key molecule involved in defining higher-order chromatin domain structures, it can be expected to also play a dominant role in defining the domain size. We, thus, hypothesize that the average size of chromatin domains is reciprocally proportional to the amount of CTCF. This leads to the following hypothesis that a CTCF reduction to 50% leads to less binding sites being occupied and correspondingly to an average increase in the domain size by a factor of 2.

The GLOBLE model can therefore predict the impact of CTCF depletion by correspondingly adapting the domain size but keeping all other parameters constant as for the control cells. The corresponding lethalities are derived from a linear-quadratic fit of the survival curve for the GFP KD control cells and using Equations (9) and (10) to assign the lethalities (see Section 4.7). These lethalities are kept constant for both conditions, i.e., control and CTCF-depleted cells, and only the frequency of iDSB and cDSB differs according to the change in the domain size. All relevant input parameters are summarized in Table 1.

As a consequence of the above mentioned concept, the α term, as defined by the lethality of iDSB according to Equation (9), should be untouched in the case of CTCF depletion, since the relative change in the number of iDSB at low doses is negligible. In contrast, the increase in the domain size should be connected with an increased β term of the survival curves according to Equation (10), since due to the constant α values, a decrease in N_D_ must be compensated by a corresponding increase in β.

### 2.5. Comparison of Model Predictions with Experimental Data

**Figure 4:**
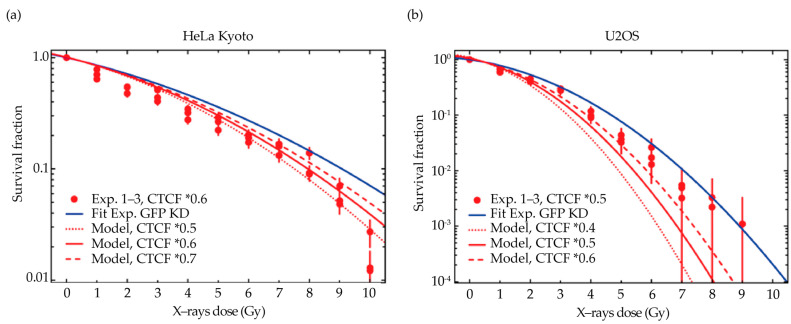
GLOBLE predictions of clonogenic survival upon different levels of CTCF depletion in HeLa Kyoto (**a**) and U2OS (**b**) cells, in comparison to the experimental data. The LQ parameters for the reference conditions were determined based on the GFP KD control survival (full blue curve, fitting weighted by $\frac{1}{relative\;error^{2}}$). Full red lines indicate the model’s predictions for the experimentally determined CTCF depletion level, whose experimental data from three biological replicates is shown as dots for comparison (whiskers = error bars); the predictions for the other knockdown levels are shown as dashed lines to indicate the expected impact of the knockdown efficiency. (**a**) GLOBLE predictions for HeLa Kyoto. (**b**) GLOBLE predictions for U2OS. The same predictions are plotted with equal y axis scaling in Figure S5c,d, for comparison.

Our model predicted that CTCF depletion results in a change in the survival curves towards increased sensitivity, as a consequence of the hypothesized increase in the chromatin domain size and the corresponding higher number of more severe cDSB. In general, the order of magnitude of the shift is in reasonable agreement with the experimental data for both cell lines (CTCF KD: red dots; GFP KD: full blue curve), as shown by the comparison with the model predictions shown in Figure 4 (model: red curves).

In particular, the expected change in sensitivity as predicted by the model is shown for different levels of CTCF depletion (full and dashed red curves). The predictions for the experimentally determined depletion level (full red curves) agree best with the experimental data of HeLa Kyoto. For U2OS cells, the predicted shift is more pronounced as compared to the measured data, which is likely due to the possible influence of the different genome sizes on the chromatin domains’ architecture. The same predictions are plotted with equal y axis scaling in Figure S5c,d, for a direct comparison of the 2 cell lines.

**Figure 5:**
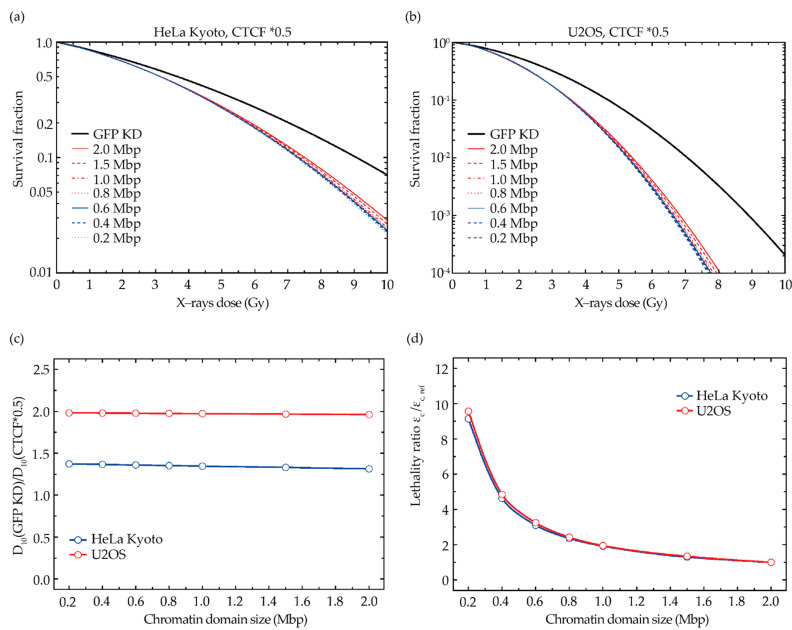
GLOBLE predictions of the radiation sensitivity analyzing the impact of CTCF depletion on differentially sized chromatin domains shows only minimal changes in a range from 2 to 0.2 Mbp. (**a**,**b**) Impact of the domain size on the predicted increase in the sensitivity after CTCF knockdown to the 50% level for HeLa Kyoto (**a**) and U2OS (**b**) cell parameters. (**c**) Corresponding change in D10 (the dose required to achieve 10% survival) as a function of the domain size. (**d**) Required change in the lethality parameter ε_c_ that describes the lethality of clustered DSB, compensating for the change in the domain size.

An important parameter of the model is the assumption of the size of the chromatin domains, which up to now was chosen to be 2 Mbp. However, since different sizes are reported for chromatin contact domains, TADs, or compartments (see the introduction for an overview), we also checked the dependence of the model prediction on the reported domain sizes (Figure 5).

Although probably somewhat counterintuitive, the predicted impact of CTCF depletion only very weakly depends on the reference domain size, as demonstrated in Figure 5 for both the HeLa Kyoto (a) and U2OS (b) cell parameters. We chose depletion to 50% of the reference CTCF level to mimic a situation with a pronounced impact of CTCF depletion for both cell lines and to compare them relatively to the potential impact of the domain size. According to Equation (3), a reduction in the domain size is connected with a correspondingly lower probability to induce cDSB, but this can be compensated for by assigning a higher lethality to each cDSB. Consequently, the relative change in the lethality resulting from an increased domain size after depletion of CTCF is very similar for different reference domain sizes (Figure 5c,d).

**Figure 6:**
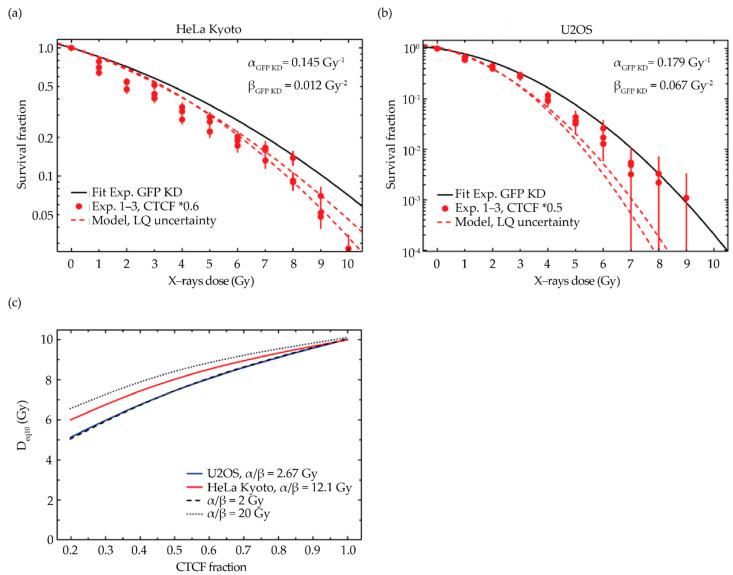
Validation of GLOBLE modeling predictions: uncertainty analysis of the linear quadratic (LQ) fitting curve and analysis of the cell line-related dependency on the impact of CTCF on radiosensitivity. (**a**,**b**) Impact of the uncertainty of the fitted LQ-parameters for the reference condition on the predicted survival curve for CTCF depletion to 60% in HeLa Kyoto (**a**) and 50% in U2OS cells (**b**), respectively. (**c**) Impact of the cellular system on the expected change in the sensitivity by CTCF depletion. Besides the model predictions for the two cell lines used in this study, additional calculations were performed for hypothetical cell lines characterized by a very low α/β-ratio of 2 Gy (α = 0.3 Gy^−1^, β = 0.015 Gy^−2^) and a high α/β-ratio of 20 Gy (α = 0.3 Gy^−1^, β = 0.0015 Gy^−2^).

Additionally, we checked the impact of the uncertainties of the fitted linear-quadratic parameters, which are used as input for the model, on the predicted change in the sensitivity after depletion of CTCF (Figure 6). Based on the errors Δα and Δβ as given in Table 1, we used 2 different combinations of either (α + Δα, β − Δβ) or (α − Δα, β + Δβ) as input parameters for the model. As shown in Figure 6a,b, the uncertainties mainly affect the high-dose part of the curves for both cell lines but otherwise do not substantially affect the agreement between the model prediction and experimental data.

Finally, we analyzed the expected effect of CTCF depletion for different cellular sensitivity characteristics (Figure 6c). In addition to the parameters representing HeLa Kyoto and U2OS cells, we chose hypothetical cell lines characterized by α/β = 2 Gy and α/β = 20 Gy. The impact of the chosen cell line is not very pronounced, but there is a systematic shift towards increasing sensitivity with a decreasing α/β-ratio (Figure 6c). This can be explained by the fact that, according to the model hypothesis, it is the β term that is affected by CTCF depletion, whereas the α term remains unaffected. Therefore, since the relative contribution of the β term increases with the decreasing α/β-ratio, the impact of CTCF depletion is also expected to be most pronounced in the case of small α/β values.

### 2.6. A CTCF Effect on Radiosensitivity Is also Observed in Mouse ES Cells and Is CTCF Dose Dependent

**Figure 7:**
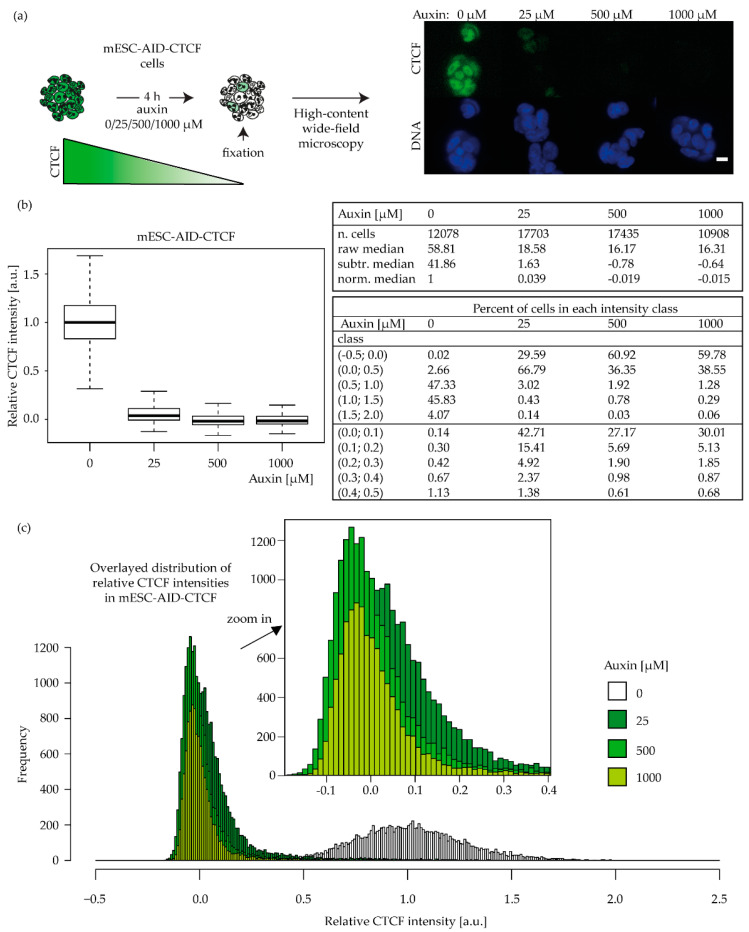
Degron system validation in mESC-AID-CTCF cells. (**a**) Cells were subjected to different concentrations (0, 25, 500, 1000 µM) of auxin for 4 h, fixed, and imaged using a high-content wide-field microscope (scale bar 10 µm, Figure S1, Table S3); (**b**,**c**) Nuclear CTCF-GFP mean intensities were measured, the background as measured in untagged mESC was subtracted, and the resulting data were normalized to the untreated control sample (0 µM); normalized CTCF values are shown as boxplots (**b**) and as frequency distributions (**c**), together with the percent of cells lying within the different classes of CTCF intensities. The results are based on four biological replicates (Table S4). See Figure S3 for the boxplot interpretation. In Figure S6, the relative kinetics of auxin-induced CTCF depletion and recovery after auxin wash-off are further investigated in a time course experiment.

The use of a mouse embryonic cell line engineered with an auxin-inducible degron for CTCF, bearing a CTCF-GFP-tag (mESC-AID-CTCF [21], Table S1), allowed us to further investigate the effects of depletion in a completely different cell system and with a more synchronous dose regulation (Figure 7a). We applied different auxin concentrations (0, 25, 500, 1000 µM; Figure 7a) for 4 h. We obtained differential levels of depletion (Figure 7b), with circa 32% residual CTCF in the 25 µM sample and 28% CTCF in the 500-µM- and 1000-µM-treated cells, compared to the untreated cells.

**Figure 8:**
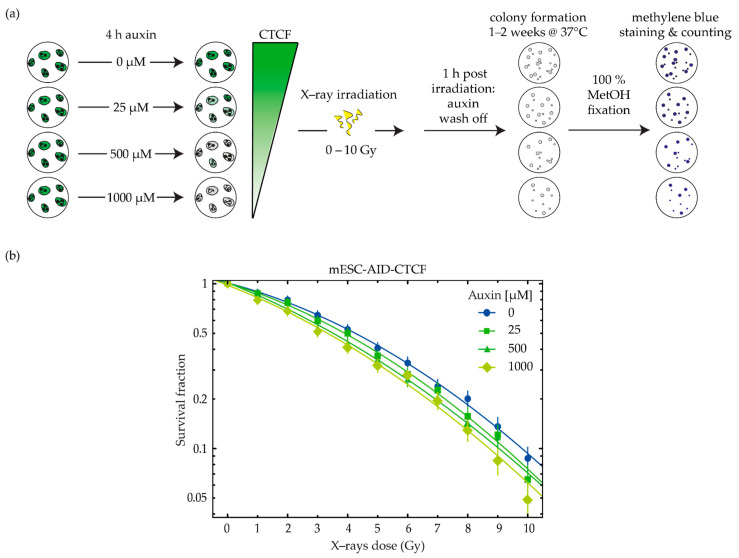
Clonogenic assay of mESC-AID-CTCF cells upon auxin-induced CTCF depletion and irradiation. (**a**) Auxin treatment was applied for 4 h (0, 25, 500, 1000 µM) after single-cell seeding, at which time point cells were irradiated with X-rays (0–10 Gy); 1 h after irradiation, auxin was washed off and single cells were allowed to form colonies for 7–10 days under standard culture conditions. Colonies were then fixed with cold 100% methanol and stained with methylene blue. The colonies with more than 50 cells were microscopically identified and counted by eye; (**b**) Each data point was normalized to the average of the respective unirradiated control and plotted as relative survival fractions in a semi-logarithmic scale with a linear-quadratic dose–response curve fitting (whiskers = error bars; fit weighted by $\frac{1}{relative\;error^{2}}$). The results consist of four biological replicates (Table S6).

When we subtracted the background GFP values measured in wild-type untagged mESC (ES14 [65], Table S1), we obtained a median 4% of CTCF left in the 25 µM sample and below 1% left in the 500 and 1000 µM samples (Figure 7b). Besides the similar median values, the 25 and 500 µM samples displayed a different CTCF intensity distribution. In the lowest auxin treatment, circa 70% of cells retained a small amount of CTCF, and in particular, 43% of the population showed a level of at least 10% of CTCF left while in 30% of cells, CTCF was not detectable. With the higher auxin treatments, the opposite was observed, with CTCF being equal to background levels in 60% of the cell population and the remaining 30% showing circa 10% residual CTCF (see “percent of cells in each intensity class” in Figure 7b,c). We next evaluated the survival of the cells with different CTCF levels to irradiation (Figure 8a). We observed that the impact of CTCF on radiosensitivity is dose dependent, as the fitting curves show decreased survival along with decreasing CTCF levels (Figure 8b, see Table S6 for survival data statistics and Figure S4 for colony formation images).

As it is reported that near to complete depletion does not allow cells to grow longer than four days [21], we decided to wash off auxin one hour after irradiation, in order to validate its impact solely on the DNA repair ability (Figure 8a). Although the samples treated with 500 and 1000 µM auxin showed an almost equivalent CTCF values distribution (Figure 7), an independent experiment was performed to assess the timing of CTCF depletion and recovery upon auxin wash off (Figure S6, Table S4). In this way, we observed that the higher the auxin concentration, the faster CTCF is depleted and the slower its recovery (Figure S6, Table S4). This explains how a slightly lower survival rate could be achieved with 1000 µM auxin (Figure 8b) even though the actual CTCF levels in the population were comparable with the 500-µM-treated sample (Figure 7). However, the decrease in survival in stem cells was lower than in the tumor cells that retained a higher CTCF level (Figure 3b,c), which may indeed be due to the faster recovery of CTCF levels in the first hour upon auxin wash off.

**Figure 9:**
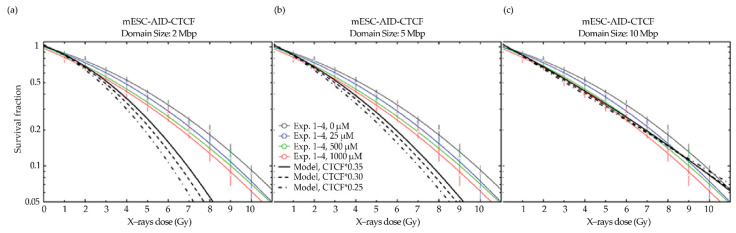
GLOBLE predictions of clonogenic survival upon different levels of auxin-induced CTCF depletion in mESC-AID-CTCF cells, in comparison to the experimental data. The reference condition (0 µM auxin) is depicted as a full thin black curve. Bold dashed black lines indicate the model predictions for the experimentally determined CTCF depletion levels, whose experimental data from four biological replicates is shown as colored fit lines (curve fit weighted by $\frac{1}{relative\;error^{2}}$) and dots (mean) for comparison (whiskers = error bars). (**a**) Predictions modeled based on the standard 2 Mbp domain size. (**b**,**c**) Predictions modeled based on a domain size of 5 (**b**) and 10 Mbp (**c**).

The modeling of CTCF depletion in the engineered cell line was performed qualitatively for residual values close to the observed raw CTCF residual values without background subtraction, i.e., 35%, 30%, and 25% (see Figure 9), for 2 reasons. On the one hand, the experimental method reaches its limits, since the detected CTCF signal is close to the background value for the higher auxin concentrations, leading to a substantial fraction of negative values in the intensity distribution (Figure 7b); therefore, the low values are subject to substantial uncertainties. On the other hand, the near-to-complete depletion imposed mathematical limits on our modeling. The model calculations based on the raw intensity values should therefore be interpreted as a lower limit for the expected impact of the CTCF depletion in this case.

When we applied our GLOBLE modeling approach based on the standard 2 Mbp domain size, the obtained model predictions deviated from the experimental data (Figure 9a), likely due to the higher kinetics of CTCF recovery upon auxin wash off (Figure S6, Table S4). The resulting predictions of survival are therefore lower than the actual experimental data. This is probably due to the much faster CTCF recovery in this degron system compared to the KD systems used earlier. However, when a higher domain size of 5 or 10 Mbp was used, the survival predictions were closer to the experimental data (Figure 9b,c). This is consistent with a unique and looser chromatin architecture and correspondingly larger domain sizes in embryonic stem cells [66].

**Figure 10:**
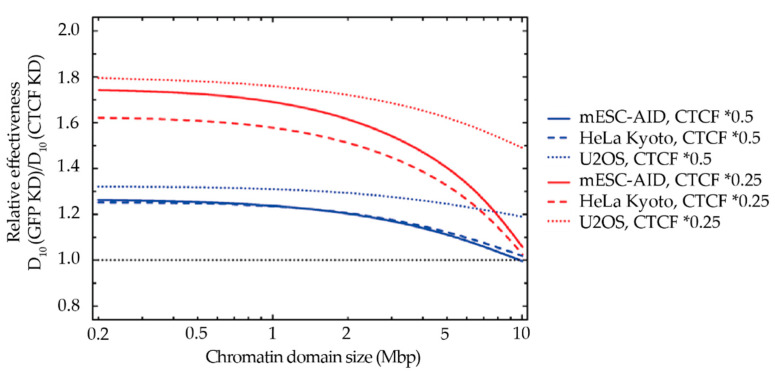
GLOBLE predictions of radiation sensitivity analyzing the impact of CTCF depletion on differentially sized control chromatin domains. As an indicator of the impact, the ratios of D10 (the dose required to achieve 10% survival) under control conditions to the D10 after CTCF depletion are used. CTCF depletion has the most significant impact for control domain sizes below approximately 2 Mbp for all 3 cell lines, whereas towards larger control domain sizes, the impact is reduced. The black dotted line indicates the reference value, corresponding to CTCF depletion having no impact.

To investigate this possibility, we analyzed the domain-size dependency on the impact of CTCF depletion as in Figure 5c, this time in a range from 0.2 to 10 Mbp (Figure 10). A CTCF depletion up to 25% or 50% residual levels similarly impacts domains of different sizes up to 2 Mbp. However, the outcome changes beyond 2 Mbp: our model predicted a substantially reduced sensitivity to CTCF depletion when the considered domains have a size closer to 5 or 10 Mbp (Figure 10). The reduction in the impact of CTCF towards larger reference domain sizes is most pronounced for mESC and HeLa cells, whereas for U2OS, the effect is considerably smaller. This can be traced back to the much smaller α/β ratio for this cell line, indicating a larger impact of clustered DSB as compared to isolated DSB and consequently also a larger impact of CTCF depletion. More insights on the possible reasons for this deviation are illustrated in Section 3.

## 3. Discussion

In this study, we tested the survival of CTCF-depleted cells treated with increasing amounts of ionizing radiation, to establish a biophysical model of the impact of CTCF on cellular radiosensitivity. The measurements of the residual CTCF amounts after esiRNA- or auxin-induced depletion (Figure 2 and Figure 7) were integrated into an adaptation of the Giant-LOop-Binary-LEsion (GLOBLE) model (see Section 4.7, [55,56]). This allowed us to qualitatively predict the decreased survival of CTCF-depleted cells, based on the higher probability of DSB clustering and the related higher lethality (Figure 4 and Figure 9).

We previously demonstrated that CTCF is essential for the correct spreading of the signaling of DNA damage, i.e., phosphorylation of the histone variant H2AX [31]. Based on our high-resolution microscopy measurements, when a single DSB occurs on a multi-loop domain, whose bases are held together by CTCF, on average, four chromatin loops within this domain are decorated by γH2AX [31]. When this domain-wide signal spreading is not possible due to reduced CTCF amounts, the following repair cascade is impaired and consequently, the survival potential too. Moreover, the reduced CTCF presence translates into diminished bordering of the chromatin domains and hence into more DSBs clustering together. As the GLOBLE model assigns a higher lethality to clustered DSBs, which affects the higher-dose part of the survival curve, we could predict a relationship between CTCF depletion and increased cell lethality upon ionizing radiation (Figure 4 and Figure 9). We hypothesize that the lower effect seen in the CTCF-depleted osteosarcoma cells compared to the cervical cancer cells (Figure 3, Figure 4, and Figure S5) reflects a fact that is often neglected: that the endogenous levels of CTCF are significantly different in the two cell lines (Figure 1). As U2OS cells exhibit approximately twice the amount of CTCF compared to HeLa Kyoto cells, residual CTCF levels after depletion in U2OS were still higher, leading to a lower impact on survival relative to the other tumor type.

**Figure 11:**
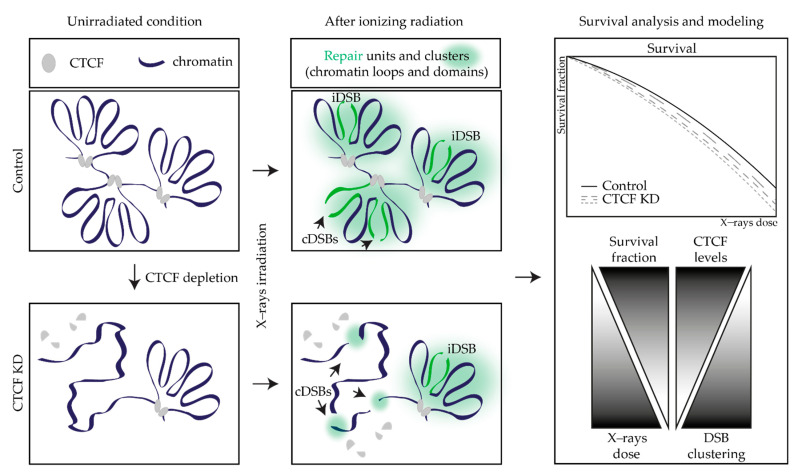
Lower CTCF levels lead to less chromatin domains and a higher probability that DSBs will not be separated into different chromatin domains (cDSBs). The cDSBs have higher lethality due to the loss of DNA, resulting in decreased survival.

Our modeling approach considered the effect of different levels of CTCF depletion on radiosensitivity, assuming an identical impact on all cell cycle phases, as cells were not synchronized, and the relative effects were averaged. The main model parameters, i.e., the lethalities assigned to iDSBs and cDSBs, were determined from fitting of the survival curves under reference conditions (see Section 4.7). Importantly, uncertainties in these parameters are small and do not substantially affect the model predictions, which means the model settings are robust (Figure 6a,b). The model predictions were built on the distribution of DSBs as being isolated or clustered together within the same chromatin domains defined as “Giant LOops” of 2 Mbp (based on [32,67,68,69,70]). However, we also modeled how CTCF depletion could differentially affect chromatin domains of different sizes and predicted that CTCF-dependent radiosensitivity does not change in a size range between 0.2 and 2 Mbp (Figure 5). In other words, the effects of CTCF depletion on such domains would equally contribute to the enhanced radiosensitivity independently of the size under reference conditions. Despite these simplistic assumptions that could indeed average different situations in vivo, our model was successful in predicting the general trend and the order of magnitude by which different CTCF levels affect the clonogenic survival curves in the two tumor cell lines under study (Figure 4). Postulation of a dependency between the presence of the chromatin domain-anchoring protein CTCF and the tendency of DSBs to cluster within a domain was sufficient to recapitulate the experimental data of both tumor cells. We can hence conclude that the dominant mechanism by which reduced CTCF amounts result in increased radiosensitivity is based on the premise that in the absence of defined domain boundaries, the probability of DSBs clustering together is enhanced (Figure 11). As the lethality of cDSBs is higher and related to the β term of the survival functions, this is reflected in the higher impact of CTCF depletion in the high-dose part of the survival curves.

We next confirmed the impact of CTCF depletion further by using mouse embryonic stem cells engineered with an auxin-inducible degron system for the depletion of CTCF (Figure 7, Table S1, [21]). Embryonic stem cells are characterized by weakly condensed heterochromatin and large nucleosome-free regions [71], which is indicative of their high transcriptional activity [72,73]. The unique chromatin architecture of stem cells consists of infrequent and primarily short-ranged loop domains, whose boundaries are more permissive of interactions and are reinforced only during differentiation [66]. As stem cells have high pan-nuclear γH2AX levels, associated with global chromatin decondensation [74] but not DSBs, they are expected to have a consistent response to DSBs and were shown to have faster DSB repair rates than somatic cells [75]. Together with the suppression of mutagenesis, the elimination of damaged cells through apoptosis appears to be a mechanism that ensures the genome integrity in pluripotent embryos ([76,77,78], as also reviewed in [79]). It is widely accepted in radiation biology that DSBs are the most genotoxic lesions (as reviewed in [80,81,82]). Their repair in somatic mammalian cells preferentially uses the non-homologous end-joining (NHEJ) pathway, which commonly occurs between G1 and early S-phase and can be error prone when the broken ends require processing before religation [52,53,83,84,85]. Since the S-phase is the predominant cell cycle phase in highly proliferative embryonic stem cells (approximately 75% of the time [86]), their repair mechanism of choice is likely the homologous recombination (HR) pathway [87,88,89], which ensures high fidelity through the use of a template and mainly occurs between late S-phase and G2 (as reviewed in [79]). The different chromatin architecture and choice between DDR pathways might be a reason why the survival of mESC-AID-CTCF cells upon CTCF depletion does not meet our model predictions (Figure 9) and also why the experimental data showed a different sensitivity between tumor and embryonic cells (Figure 3 and Figure 8, respectively). Indeed, stem cells are more sensitive to damage, but they readily eliminate damaged cells and those that do not undergo apoptosis likely have higher survival potential and successfully proliferate into colonies. On the other hand, the tumor cell lines under study would accumulate important damage and show less survival in the long term. Moreover, the lethality of CTCF depletion [11,12,13] is required to timely limit the depletion. This could not be taken into account in the modeling, which therefore predicts what would happen after longer CTCF depletion (Figure 9).

Mechanistic insights into the decreased survival of CTCF-depleted cells are found in the assumptions of the GLOBLE model itself and the literature (see Section 2.4 and Section 4.7). The modeling approach presented here predicts cell survival based on the reverse proportion between the amounts of CTCF and the domain size, with the latter instead being proportional to the probability of cDSBs occurring (Figure 11). More than the induced number of DSBs, previous studies attributed cellular death to the delayed DSB rejoining and retained persistent damage at later time points [67,69,70,90,91,92,93], often related to nano- [94,95,96,97] or micrometer [68,98,99,100] clustering of the initial lesions. The yields of DSB induction considered in the model implicitly include not only the prompt DSBs but also those originating from clustered non-DSB lesions [69,70,101,102,103,104].

In previous studies based on filter elution techniques and flow cytometry detection of γH2AX [69,70], the GLOBLE model could reproduce the gradually slower processing of DSBs after high doses, supporting the higher toxicity of clustered DSBs at the micrometer scale in higher-order chromatin structures (extensively discussed in [64,69,70]). Our model distinguishes iDSB from cDSB, where a cluster is defined by a domain containing 2 or more DSBs (see Section 4.7). Even when the distance between two DSBs within the same chromatin domain is so high that these can be considered as single DSBs in terms of their individual repair, inter-DSBs DNA become a spatially independent fragment and any stabilization of its ends is at risk. A further development of our model could make another distinction within the cDSB class to predict in more detail the impact of DSB density on lethality. On the one hand, the more DSBs accumulate within a chromatin domain, the more DNA is fragmented, leading to a complex task for the repair machinery. On the other hand, two DSBs very close to the bases of a domain would evict a large portion of DNA, and additional fragmentation would not further enhance the damage severity. This distinction would hence allow us to define what is more critical for repair, the DSB density, defined as the ratio between the domain size and the number of DSBs within the domain, or the domain size itself as a self-determinant of the length of DNA that can be deleted with two DSBs at its bases. Moreover, our modeling could be additionally refined with differentiation of chromatin states into eu- or heterochromatin and their relative impact on domain sizes, and by considering the activity of different proteins and transcription events.

As previously mentioned, DSBs are considered the key damage in the field of radiation biology, but ionizing radiation induces various types of DNA lesions other than DSBs [26,27,28]. However, these occur both in control and in CTCF-depleted cells and it is unlikely that the deviations seen under CTCF depletion conditions could be mainly traced back to other damage, such as SSB or crosslinks. Damage to lipids and proteins is also a consequence of ionizing radiation, but the related effects will become visible only at extremely high doses due to the high cellular numbers of these molecules. At the dose ranges typically used for cell survival studies (0–10 Gy), these effects can be considered negligible as compared to damage to DNA.

Interestingly, the dose dependency found here between CTCF and radiosensitivity is consistent with the observation of Nora and colleagues on chromatin folding [21]. They observed that chromatin changes scaled together with CTCF depletion, with insulation of domains being more preserved in cells with 15% residual CTCF than in those cells with almost complete depletion [21]. Nonetheless, it is worth noting that even if TADs are lost upon CTCF depletion, higher-order A/B compartments are largely unaffected [21]. This might partially explain the overall small decrease in the survival of depleted cells and points to the fact that our understanding of the chromatin organization and related repair is still incipient.

## 4. Materials and Methods

### 4.1. Cell Culture

HeLa Kyoto and U2OS cells (Table S1) were grown at 37 °C in a humidified atmosphere with 5% CO_2_ and cultured in Dulbecco’s modified Eagle’s medium (DMEM) high glucose (Cat. No.: D6429, Sigma-Aldrich Chemie GmbH, Steinheim, Germany) supplemented with 50 µg/mL gentamicin, 20 mM L-glutamine (Cat. No.: G7513, Sigma-Aldrich Chemie GmbH, Steinheim, Germany), and 10% fetal calf serum (Cat. No.: F7524, Sigma-Aldrich Chemie GmbH, Steinheim, Germany).

mESC-AID-CTCF and wild-type ES-E14TG2a cells (Table S1) were cultured in Dulbecco’s modified Eagle’s medium (DMEM) high glucose (Cat. No.: D6429, Sigma-Aldrich Chemie GmbH, Steinheim, Germany) supplemented with 15% fetal calf serum (Cat. No.: F7524, Sigma-Aldrich Chemie GmbH, Steinheim, Germany), 1× non-essential amino acids (Cat. No.: M7145, Sigma-Aldrich Chemie GmbH, Steinheim, Germany), 1× penicillin/streptomycin (Pen/Strep) (Cat. No.: P4333, Sigma-Aldrich Chemie GmbH, Steinheim, Germany), 1× L-glutamine (Cat. No.: G7513, Sigma-Aldrich Chemie GmbH, Steinheim, Germany), 0.1 mM beta-mercaptoethanol (Cat. No.: 4227, Carl Roth, Karlsruhe, Germany), 1000 U/mL recombinant mouse LIF (Millipore), and 2i (1 M PD032591 and 3 M CHIR99021 (Cat. Nos.: 1408 and 1386, respectively, Axon Medchem, Netherlands)) on gelatin-coated culture dishes (0.2% gelatin/ddH_2_O; Cat. No.: G2500, Sigma-Aldrich Chemie GmbH, Steinheim, Germany).

### 4.2. CTCF Knockdown

An esiRNA pool against human CTCF (Table S5; Cat. No.: EHU130111, MISSION^®^ esiRNA, Sigma-Aldrich Chemie GmbH, Steinheim, Germany) was used to deplete HeLa Kyoto and U2OS cells from the protein. The pool targets the 692–1195 region of the human CTCF transcript (NM_006565.3). Both cancer cell lines were transfected with 15 nM of esiRNA against either human CTCF or EGFP as a mock-depleted control (Table S5; Cat. No.: EHUEGFP, MISSION^®^ esiRNA, Sigma-Aldrich Chemie GmbH, Steinheim, Germany) using the electroporation Neon™ Transfection System (Cat. No.: MPK5000, Thermo Fisher Scientific, Waltham, MA, USA). Voltage, width, and pulse for the different cell lines were applied as follows: HeLa Kyoto 1005 V, 35 ms, 2×; U2OS 1230 V, 10 ms, 4×. For the knockdown validation (Figure 2), cells were seeded on coverslips immediately after transfection and fixed with 3.7% formaldehyde/1× phosphate-buffered saline PBS (Cat. No.: F8775, Sigma-Aldrich Chemie GmbH, Steinheim, Germany) at different time points (24, 48, 72 h). The results shown in Figure 2 are based on 3 biological replicates (Table S4). The same HeLa Kyoto and U2OS transfection aliquots were diluted in parallel for single-cell seeding to perform the clonogenic assay (Figure 3, Section 4.6). For CTCF depletion and its validation with the degron system (Figure 7), mESC-AID-CTCF cells were seeded on gelatinized coverslips and treated with auxin-supplemented medium (0, 25, 500, 1000 μM; 3-Indoleacetic acid IAA, Cat. No.: I3750, Sigma-Aldrich Chemie GmbH, Steinheim, Germany) for 4 h, and then washed and fixed with 3.7% formaldehyde/1× PBS (Cat. No.: F8775, Sigma-Aldrich Chemie GmbH, Steinheim, Germany). The results shown in Figure 7 are based on 4 biological replicates (Table S4). The same auxin- supplemented medium was given in parallel to the single cells seeded for the clonogenic assay (Figure 8; Section 4.6). In an independent time course experiment (3 biological replicates; Table S4; Figure S6), stem cells seeded on gelatinized coverslips (0.2% gelatin/ddH_2_O; Cat. No.: G2500, Sigma-Aldrich Chemie GmbH, Steinheim, Germany) were treated with 25, 500, and 1000 μM auxin and fixed with 3.7% formaldehyde/1× PBS (Cat. No.: F8775, Sigma-Aldrich Chemie GmbH, Steinheim, Germany) at different time points of auxin treatment (0.5, 1, 2, 3, and 4 h), washed 3 × with warm 1× PBS after 4 h, replaced with fresh medium, and CTCF recovery allowed until 24 h. Multiple time points were measured after auxin wash off (0.5, 1, 2, 4, 6, 8, 18, and 24 h).

### 4.3. CTCF Immunostaining

The previously fixed HeLa Kyoto and U2OS cells were washed with 1× PBS, permeabilized 15′ with 0.7% Triton^™^ X-100 (Cat. No.: T8787, Sigma-Aldrich Chemie GmbH, Steinheim, Germany) and washed again prior to 30′ blocking in 1% BSA (Cat. No.: A4503, Sigma-Aldrich Chemie GmbH, Steinheim, Germany). Incubation overnight at 4 °C with the primary rabbit anti-CTCF antibody followed. Information on the antibodies is listed in Table S2. Cells were then washed 2× with 1× PBS for 5′ and 3 × with 0.01% Tween^®^ 20 (Cat. No.: 9127.1, Carl Roth, Karlsruhe, Germany). The secondary goat anti-rabbit IgG (H + L) AF594 antibody was incubated for 1 h at room temperature, followed by washing as described above. DNA was counterstained with 10 µg/mL DAPI (4′,6-diamidino-2-phenylindole, Cat. No.: D27802, Sigma-Aldrich Chemie GmbH, Steinheim, Germany) for 10′ at room temperature, cells dipped in ddH_2_O, and mounted with Vectashield^®^ antifade medium (Cat. No.: NC9265087, Thermo Fisher Scientific, Walham, MA, USA) on coverslides. All dilutions mentioned above were performed in 1× PBS, except antibody dilutions in 1% BSA/1× PBS. mESC-AID-CTCF cells and wild-type ES-E14TG2a cells were only subjected to permeabilization, DNA counterstaining, and mounting as described above.

### 4.4. CTCF Western Blot

Cells were washed with ice-cold 1× PBS and collected using a cell scraper. Upon centrifugation at 500× *g* for 5′, cells were lysed for 1 h at 4 °C in lysis buffer (150 mM NaCl, 200 mM Tris pH 8.0, 5 mM EDTA) with 0.4% NP-40, protease, and phosphatase inhibitors (1 mM PMSF, 20 μM PepA, 20 mM NaF, 100 mM Na_3_VO_4_). Lysates were then centrifuged at 13,000 rpm for 20′ at 4 °C and supernatant collected. Protein concentrations were measured using the protein bovine serum albumin standard assay kit (Cat. No.: 23208, Thermo Fisher Scientific, Walham, MA, USA) according to the manufacturer’s protocol. Increasing amounts of protein lysates (30, 40, and 50 μg) were loaded along with the protein standard ladder (Cat. No.: P7719S, New England Biolabs, Ipswich, MA, USA) on a 6% polyacrylamide gel. This was followed by electrophoresis for 1.5 h in ice-cold 1× Laemmli electrophoresis running buffer (25 mM Tris base, 192 mM glycine, 3.5 mM SDS). Protein transfer to a 0.2 μm nitrocellulose membrane (Cat. No.: 162-0112, Bio-Rad Laboratories, Hercules, CA, USA) was performed in 1× transfer buffer (Pierce™ Western Blot Transfer Buffer 10 ×, Cat. No.: 35045, Thermo Fisher Scientific, Walham, MA, USA) using a semi-dry transfer system (Cat. No.: 1703940, Trans-Blot^®^ SD Semi-Dry Transfer Cell, Bio-Rad Laboratories, Hercules, CA, USA) for 55′ at 25 V. The membrane was subsequently blocked in 5% low-fat milk for 30′. Incubation with primary and secondary antibodies was performed in 5% milk at 4 °C overnight and at room temperature for 1 h, respectively. Each incubation was followed by 3 × 10′ washing with 0.075% Tween^®^ 20 in 1× PBS (Cat. No.: 9127.1, Carl Roth, Karlsruhe, Germany) and signals were detected using the Amersham™ Imager 600 (GE Healthcare, Chicago, IL, USA). Analysis was performed with Image Lab Software, Version 6.1 (SOFT-LIT-170-9690-ILSMAC-V-6-1, Bio-Rad Laboratories, Hercules, CA, USA). The antibodies and imaging conditions are listed in Tables S2 and S3, respectively.

### 4.5. Microscopy Imaging and Quantification

The samples were imaged with a high-content wide-field microscopy system Operetta^®^ and images were analyzed with the Harmony^™^ software (both PerkinElmer, see Table S3), as described in Figure S1. In particular, the DAPI signal was used to create a nuclear mask and select nuclei. The nuclear intensities of both DAPI—DNA and AF594—CTCF or GFP—CTCF signals were measured and exported. The results tables were analyzed in RStudio (Version 0.99.902—© 2009–2016 RStudio Inc., Boston, USA) to produce the plots shown in Figure 1, Figure 2, Figure 7 and Figures S3 and S5. See Figure S3 for the boxplot interpretation.

### 4.6. Clonogenic Assay

HeLa Kyoto and U2OS cells were irradiated with X-ray doses of 0–10 Gy (1 Gy/58 s, 250 kV, 10 mA; Isovolt Titan, GE Sensing & Inspection Technologies, Ahrensburg, Germany) at 24 h post esiRNA transfection (Figure 3), at the respective time point of maximum depletion for each cell line (Figure 2b,c). Cells were incubated with 5% CO_2_ at 37 °C for 7–10 days until colonies formed. Colonies were then fixed with cold 100% methanol and stained with 0.2% methylene blue in 50% methanol/1× PBS followed by washing in water (Figure 3a). The colonies with more than 50 cells were verified by microscopy and counted by eye. The results for both HeLa Kyoto and U2OS consist of three biological replicates, each composed of technical replicates (Table S6). mESC-AID-CTCF cells were treated with different auxin concentrations (0, 25, 500, and 1000 μM; 3-Indoleacetic acid IAA, Cat. No.: I3750, Sigma-Aldrich Chemie GmbH, Steinheim, Germany) for 4 h prior to irradiation (the same conditions as above), washed 3 times with 1× PBS, and replaced with fresh medium 1 h after irradiation (Figure 8a). Cells were then allowed to form colonies and treated as described above for the tumor cell lines. The results for mESC-AID-CTCF cells consist of four biological replicates (Table S6). The number of colonies was plotted as a survival ratio, in which each value was normalized to the average of the respective unirradiated controls.

The homegrown “gd” software (http://bio.gsi.de/DOCS/gd.htm, accessed on 10 March 2022; GSI Helmholtzzentrum für Schwerionenforschung GmbH, Biophysics Department, Darmstadt, Germany) [105] was used to plot the survival curves shown in Figure 3, 8, and S5. A linear-quadratic dose–response curve fitting weighted by $\frac{1}{relative\;error^{2}}$ was applied (dots = mean; whiskers = error bars; see the footer of Table S6 for the calculation of the relative error).

### 4.7. Modeling of Cell Survival Using the Giant-Loop Binary Lesion (GLOBLE) Model

For standard applications of the GLOBLE model, the domains are assumed to contain a genomic content of approximately g_D_ = 2 Mbp, covering a volume with an approximate side length of L_D_ = 0.5 μm in a cell nucleus with a typical DNA content of G = 6 Gbp and a cell nuclear volume of V_Nuc_ = 500 μm^3^. The number of domains is then given by:

(1) $$N_{D}=\frac{G}{g_{D}}$$

resulting in *N_D_* = 3000 domains per nucleus for the reference conditions given above. For cells with different genome sizes G, the total volume and the number of domains are correspondingly scaled, keeping the volume and side length and genomic content of the individual domains constant.

Photon radiation produces DSBs that are randomly distributed throughout the genome, with a yield of *Y_DSB_* = 30 DSB/Gy/cell. Therefore, the mean number of *DSB*s produced by a given dose *D* in the whole cell nucleus is:

(2) $$\bar{N_{DSB}\left(D\right)}\;=Y_{DSB}\cdot D$$

and the mean number of *DSB* induced per domain is:

(3) $$\bar{n_{DSB}\left(D\right)}=\frac{\bar{N_{DSB}\left(D\right)}}{N_{D}}$$

The probability of inducing isolated DSB (iDSB), characterized by exactly 1 DSB in a domain, or clustered DSB (cDSB), characterized by 2 or more DSB in a domain, is determined by randomly distributing DSB within the nucleus, based on a yield Y_DSB_ of 30 DSB/Gy/cell nucleus and evaluating the Poisson statistics for the corresponding mean value of DSB induced in a domain at a given dose D:

(4) $$P_{DSB}\left(k,\bar{n}\right)=\frac{{\bar{n}}^{k}}{k!}e^{-\bar{n}}$$

Therefore, the number of iDSB and cDSB is given by:

(5) $$n_{i}\left(D\right)=P_{DSB}\left(1,\bar{n}\right)\cdot N_{D}=\bar{n}e^{-\bar{n}}\cdot N_{D}$$

(6) $$n_{c}\left(D\right)=(1-P_{DSB}\left(0,\bar{n}\right)-P_{DSB}\left(1,\bar{n}\right))\cdot N_{D}$$

No further distinction according to the specific number of DSB contained in a cDSB is made, i.e., cases with just 2 DSB are handled identically to cases with more than 2 DSB.

Different lethalities ε_i_ and ε_c_ are assigned to the classes of iDSB and cDSB, and the final effect on cell kill at a given dose D is determined by multiplying the number of damages in each class, n_i_ and n_c_, with the corresponding lethalities and finally summing it:

(7) $$S\left(D\right)=e^{-\left(n_{i}\left(D\right)\varepsilon_{i}+n_{c}\left(D\right)\varepsilon_{c}\right)}$$

The lethalities ε_i_ and ε_c_ are related to the parameters α and β of the standard LQ-formulation of the survival curves:

(8) $$S\left(D\right)=e^{-\left(\alpha D+\beta D^{2}\right)}$$

in the following way:

(9) $$\varepsilon_{i}=\frac{\alpha }{\alpha_{DSB}}$$

(10) $$\varepsilon_{c}=2\left(\frac{N_{D}\beta +\alpha_{DSB}\alpha }{\alpha_{DSB}^{2}}\right)$$

with N_D_ being the number of domains per cell nucleus and α_DSB_ representing the yield of DSB per Gy. Therefore, ε_i_ is uniquely linked to the initial slope of the dose–response curve as given by the linear term α, and ε_c_ is defined by a mixture of the contributions from the linear and the quadratic term β.

The main assumption for the application of the model to describe the impact of CTCF depletion is that with the decrease in CTCF, the domain size correspondingly increases. For example, if the fraction of CTCF remaining after depletion is given by *f_CTCF_,* the modified domain size is given by:

(11) $$g_{D}^{\prime }=\frac{1}{f_{CTCF}}g_{D\;}$$

The number of domains correspondingly decreases, i.e.,:

(12) $$N_{D}^{\prime }=f_{CTCF}\cdot N_{D\;}$$

The modified numbers of iDSB and cDSB are then determined as described in Equations (1)–(6), but the lethalities of iDSB and cDSB are kept identical to those under reference conditions. In principle, the genome size could also be expected to affect the sensitivity, since N_D_ increases with increasing DNA content when assuming a constant domain size. However, through calibration of the model by means of the experimental data for cells with a normal CTCF content, any changes in the DNA content are compensated for by corresponding changes in the lethality parameters (at least within the typical range of DNA content values for mammalian cells). GLOBLE model calculations were applied and the homegrown “gd” software (http://bio.gsi.de/DOCS/gd.html, accessed on 10 March 2022; GSI Helmholtzzentrum für Schwerionenforschung GmbH, Biophysics Department, Darmstadt, Germany) was used to produce the plots shown in Figure 4, Figure 5, Figure 6, Figure 9, Figure 10 and Figure S5. The GLOBLE parameters are listed in Table 1. The linear-quadratic parameters α and β and the corresponding lethalities ε_i_ and ε_c_ were determined from the survival curves under GFP KD conditions (curve fit weighted by $\frac{1}{relative\;error^{2}}$), after pooling all respective biological replicates (3 biological replicates of HeLa Kyoto and U2OS, 4 biological replicates of mESC-AID-CTCF cells, Table S6). A simple implementation of the model as an Excel sheet is available in the materials; a screenshot of the Excel sheet is shown in Figure S7.

## 5. Conclusions

Overall, we confirmed that the survival potential of different cell lines after exposure to ionizing radiation is dependent on CTCF. We showed that survival is finely tuned proportional to the CTCF dosage, which is consistent with the reported CTCF dose scaling of chromatin domains. The application of our GLOBLE modeling approach to the scenario of CTCF depletion allowed us to gain mechanistic insights relative to the decreased survival. CTCF anchoring activity limits the clustering of multiple DSBs, whose repair often has a dramatic outcome and ends with irreversible domain fractionation. Future research in cancer treatment should take into consideration the relative amount of this architectural protein in the particular tumor cell line under study and apply this model in order to predict the tumor survival after radiation therapy.

## Figures and Tables

**Table 1:** Parameters that were used in the GLOBLE model for prediction of the impact of CTCF depletion. The linear-quadratic parameters α and β and the corresponding lethalities ε_i_ and ε_c_ were determined from the survival curves under GFP KD conditions, after pooling all biological replicates. The genome size of Hela Kyoto was taken from [60] and that of U2OS estimated from that value multiplied by the genome size ratio in Figure 1b. The genome size for mouse mESC-AID-CTCF cells was taken from [63] (see Section 2.6). The default domain size was chosen to be identical for all cell lines.

Cell Line	HeLa Kyoto	U2OS	mESC-AID-CTCF
Genome size [Mbp]	9.7 × 10^3^	11.93 × 10^3^	5.2× 10^3^
Domain size [Mbp]	2	2	2
α [Gy^−1^]	0.145± 0.014	0.179 ± 0.034	0.111 ± 0.025
β [Gy^−2^]	0.012± 0.002	0.067 ± 0.0067	0.013 ± 0.0029
α/β [Gy]	12.1	2.67	8.54
ε_i_	0.0030	0.0030	0.0043
ε_c_	0.0555	0.2310	0.1085

## Data Availability

Data is available at TUdatalib (https://tudatalib.ulb.tu-darmstadt.de/handle/tudatalib/1126, accessed on 10 March 2022).

## Supplementary Materials

The following are available online at https://www.mdpi.com/article/10.3390/ijms23073896/s1.

## References

[1] Lobanenkov V.V., Nicolas R.H., Adler V.V., Paterson H., Klenova E.M., Polotskaja A.V., Goodwin G.H. (1990). A novel sequence-specific DNA binding protein which interacts with three regularly spaced direct repeats of the CCCTC-motif in the 5′-flanking sequence of the chicken c-myc gene. Oncogene.

[2] Klenova E.M., Nicolas R.H., Paterson H.F., Carne A.F., Heath C.M., Goodwin G.H., Neiman P.E., Lobanenkov V.V. (1993). CTCF, a conserved nuclear factor required for optimal transcriptional activity of the chicken c-myc gene, is an 11-Zn-finger protein differentially expressed in multiple forms. Mol. Cell. Biol..

[3] Filippova G.N., Fagerlie S., Klenova E.M., Myers C., Dehner Y., Goodwin G., Neiman P.E., Collins S.J., Lobanenkov V.V. (1996). An exceptionally conserved transcriptional repressor, CTCF, employs different combinations of zinc fingers to bind diverged promoter sequences of avian and mammalian c-myc oncogenes. Mol. Cell. Biol..

[4] Phillips J.E., Corces V.G. (2009). CTCF: Master weaver of the genome. Cell.

[5] Merkenschlager M., Nora E.P. (2016). CTCF and cohesin in genome folding and transcriptional gene regulation. Annu. Rev. Genom. Hum. Genet..

[6] Fudenberg G., Imakaev M., Lu C., Goloborodko A., Abdennur N., Mirny L.A. (2016). Formation of chromosomal domains by loop extrusion. Cell Rep..

[7] Barutcu A.R., Fritz A.J., Zaidi S.K., van Wijnen A.J., Lian J.B., Stein J.L., Nickerson J.A., Imbalzano A.N., Stein G.S. (2016). C-ing the Genome: A Compendium of Chromosome Conformation Capture Methods to Study Higher-Order Chromatin Organization. J. Cell. Physiol..

[8] Rao S.S.P., Huntley M.H., Durand N.C., Stamenova E.K., Bochkov I.D., Robinson J.T., Sanborn A.L., Machol I., Omer A.D., Lander E.S. (2014). A 3D map of the human genome at kilobase resolution reveals principles of chromatin looping. Cell.

[9] Dixon J.R., Selvaraj S., Yue F., Kim A., Li Y., Shen Y., Hu M., Liu J.S., Ren B. (2012). Topological domains in mammalian genomes identified by analysis of chromatin interactions. Nature.

[10] Lieberman-Aiden E., van Berkum N.L., Williams L., Imakaev M., Ragoczy T., Telling A., Amit I., Lajoie B.R., Sabo P.J., Dorschner M.O. (2009). Comprehensive mapping of long-range interactions reveals folding principles of the human genome. Science.

[11] Splinter E., Heath H., Kooren J., Palstra R.-J., Klous P., Grosveld F., Galjart N., de Laat W. (2006). CTCF mediates long-range chromatin looping and local histone modification in the beta-globin locus. Genes Dev..

[12] Heath H., Ribeiro de Almeida C., Sleutels F., Dingjan G., van de Nobelen S., Jonkers I., Ling K.-W., Gribnau J., Renkawitz R., Grosveld F. (2008). CTCF regulates cell cycle progression of alphabeta T cells in the thymus. EMBO J..

[13] Moore J.M., Rabaia N.A., Smith L.E., Fagerlie S., Gurley K., Loukinov D., Disteche C.M., Collins S.J., Kemp C.J., Lobanenkov V.V. (2012). Loss of maternal CTCF is associated with peri-implantation lethality of Ctcf null embryos. PLoS ONE.

[14] Zuin J., Dixon J.R., van der Reijden M.I.J.A., Ye Z., Kolovos P., Brouwer R.W.W., van de Corput M.P.C., van de Werken H.J.G., Knoch T.A., van IJcken W.F.J. (2014). Cohesin and CTCF differentially affect chromatin architecture and gene expression in human cells. Proc. Natl. Acad. Sci. USA.

[15] Schwarzer W., Abdennur N., Goloborodko A., Pekowska A., Fudenberg G., Loe-Mie Y., Fonseca N.A., Huber W., Haering C.H., Mirny L. (2017). Two independent modes of chromatin organization revealed by cohesin removal. Nature.

[16] Rao S.S.P., Huang S.-C., Glenn St Hilaire B., Engreitz J.M., Perez E.M., Kieffer-Kwon K.-R., Sanborn A.L., Johnstone S.E., Bascom G.D., Bochkov I.D. (2017). Cohesin loss eliminates all loop domains. Cell.

[17] Wutz G., Várnai C., Nagasaka K., Cisneros D.A., Stocsits R.R., Tang W., Schoenfelder S., Jessberger G., Muhar M., Hossain M.J. (2017). Topologically associating domains and chromatin loops depend on cohesin and are regulated by CTCF, WAPL, and PDS5 proteins. EMBO J..

[18] Bintu B., Mateo L.J., Su J.-H., Sinnott-Armstrong N.A., Parker M., Kinrot S., Yamaya K., Boettiger A.N., Zhuang X. (2018). Super-resolution chromatin tracing reveals domains and cooperative interactions in single cells. Science.

[19] Cremer M., Brandstetter K., Maiser A., Rao S.S.P., Schmid V.J., Guirao-Ortiz M., Mitra N., Mamberti S., Klein K.N., Gilbert D.M. (2020). Cohesin depleted cells rebuild functional nuclear compartments after endomitosis. Nat. Commun..

[20] Nishimura K., Fukagawa T., Takisawa H., Kakimoto T., Kanemaki M. (2009). An auxin-based degron system for the rapid depletion of proteins in nonplant cells. Nat. Methods.

[21] Nora E.P., Goloborodko A., Valton A.-L., Gibcus J.H., Uebersohn A., Abdennur N., Dekker J., Mirny L.A., Bruneau B.G. (2017). Targeted Degradation of CTCF Decouples Local Insulation of Chromosome Domains from Genomic Compartmentalization. Cell.

[22] Nuebler J., Fudenberg G., Imakaev M., Abdennur N., Mirny L.A. (2018). Chromatin organization by an interplay of loop extrusion and compartmental segregation. Proc. Natl. Acad. Sci. USA.

[23] Agarwal H., Reisser M., Wortmann C., Gebhardt J.C.M. (2017). Direct Observation of Cell-Cycle-Dependent Interactions between CTCF and Chromatin. Biophys. J..

[24] Hansen A.S., Cattoglio C., Darzacq X., Tjian R. (2018). Recent evidence that TADs and chromatin loops are dynamic structures. Nucleus.

[25] Mamberti S., Cardoso M.C. (2020). Are the processes of DNA replication and DNA repair reading a common structural chromatin unit?. Nucleus.

[26] Borrego-Soto G., Ortiz-López R., Rojas-Martínez A. (2015). Ionizing radiation-induced DNA injury and damage detection in patients with breast cancer. Genet. Mol. Biol..

[27] Santivasi W.L., Xia F. (2014). Ionizing radiation-induced DNA damage, response, and repair. Antioxid. Redox Signal..

[28] Huang R.-X., Zhou P.-K. (2020). DNA damage response signaling pathways and targets for radiotherapy sensitization in cancer. Signal Transduct. Target. Ther..

[29] Vignard J., Mirey G., Salles B. (2013). Ionizing-radiation induced DNA double-strand breaks: A direct and indirect lighting up. Radiother. Oncol..

[30] Schipler A., Iliakis G. (2013). DNA double-strand-break complexity levels and their possible contributions to the probability for error-prone processing and repair pathway choice. Nucleic Acids Res..

[31] Natale F., Rapp A., Yu W., Maiser A., Harz H., Scholl A., Grulich S., Anton T., Hörl D., Chen W. (2017). Identification of the elementary structural units of the DNA damage response. Nat. Commun..

[32] Rogakou E.P., Pilch D.R., Orr A.H., Ivanova V.S., Bonner W.M. (1998). DNA double-stranded breaks induce histone H2AX phosphorylation on serine 139. J. Biol. Chem..

[33] Rogakou E.P., Boon C., Redon C., Bonner W.M. (1999). Megabase chromatin domains involved in DNA double-strand breaks in vivo. J. Cell Biol..

[34] Fernandez-Capetillo O., Chen H.-T., Celeste A., Ward I., Romanienko P.J., Morales J.C., Naka K., Xia Z., Camerini-Otero R.D., Motoyama N. (2002). DNA damage-induced G2-M checkpoint activation by histone H2AX and 53BP1. Nat. Cell Biol..

[35] Lees-Miller S.P., Sakaguchi K., Ullrich S.J., Appella E., Anderson C.W. (1992). Human DNA-activated protein kinase phosphorylates serines 15 and 37 in the amino-terminal transactivation domain of human p53. Mol. Cell. Biol..

[36] Karlsson K.H., Stenerlöw B. (2004). Focus formation of DNA repair proteins in normal and repair-deficient cells irradiated with high-LET ions. Radiat. Res..

[37] Leatherbarrow E.L., Harper J.V., Cucinotta F.A., O’Neill P. (2006). Induction and quantification of gamma-H2AX foci following low and high LET-irradiation. Int. J. Radiat. Biol..

[38] Bakkenist C.J., Kastan M.B. (2003). DNA damage activates ATM through intermolecular autophosphorylation and dimer dissociation. Nature.

[39] Uziel T., Lerenthal Y., Moyal L., Andegeko Y., Mittelman L., Shiloh Y. (2003). Requirement of the MRN complex for ATM activation by DNA damage. EMBO J..

[40] Lee J.-H., Paull T.T. (2005). ATM activation by DNA double-strand breaks through the Mre11-Rad50-Nbs1 complex. Science.

[41] Singleton B.K., Torres-Arzayus M.I., Rottinghaus S.T., Taccioli G.E., Jeggo P.A. (1999). The C terminus of Ku80 activates the DNA-dependent protein kinase catalytic subunit. Mol. Cell. Biol..

[42] Sibanda B.L., Chirgadze D.Y., Ascher D.B., Blundell T.L. (2017). DNA-PKcs structure suggests an allosteric mechanism modulating DNA double-strand break repair. Science.

[43] Blackford A.N., Jackson S.P. (2017). ATM, ATR, and DNA-PK: The Trinity at the Heart of the DNA Damage Response. Mol. Cell.

[44] Zou L., Elledge S.J. (2003). Sensing DNA damage through ATRIP recognition of RPA-ssDNA complexes. Science.

[45] Wang X., Ran T., Zhang X., Xin J., Zhang Z., Wu T., Wang W., Cai G. (2017). 3.9 Å structure of the yeast Mec1-Ddc2 complex, a homolog of human ATR-ATRIP. Science.

[46] Paull T.T. (2015). Mechanisms of ATM activation. Annu. Rev. Biochem..

[47] Menolfi D., Zha S. (2020). ATM, ATR and DNA-PKcs kinases-the lessons from the mouse models: Inhibition ≠ deletion. Cell Biosci..

[48] Paull T.T., Rogakou E.P., Yamazaki V., Kirchgessner C.U., Gellert M., Bonner W.M. (2000). A critical role for histone H2AX in recruitment of repair factors to nuclear foci after DNA damage. Curr. Biol..

[49] Redon C., Pilch D., Rogakou E., Sedelnikova O., Newrock K., Bonner W. (2002). Histone H2A variants H2AX and H2AZ. Curr. Opin. Genet. Dev..

[50] Bhogal N., Jalali F., Bristow R.G. (2009). Microscopic imaging of DNA repair foci in irradiated normal tissues. Int. J. Radiat. Biol..

[51] Schultz L.B., Chehab N.H., Malikzay A., Halazonetis T.D. (2000). p53 binding protein 1 (53BP1) is an early participant in the cellular response to DNA double-strand breaks. J. Cell Biol..

[52] Scully R., Panday A., Elango R., Willis N.A. (2019). DNA double-strand break repair-pathway choice in somatic mammalian cells. Nat. Rev. Mol. Cell Biol..

[53] Swift M.L., Beishline K., Flashner S., Azizkhan-Clifford J. (2021). DSB repair pathway choice is regulated by recruitment of 53BP1 through cell cycle-dependent regulation of Sp1. Cell Rep..

[54] Hilmi K., Jangal M., Marques M., Zhao T., Saad A., Zhang C., Luo V.M., Syme A., Rejon C., Yu Z. (2017). CTCF facilitates DNA double-strand break repair by enhancing homologous recombination repair. Sci. Adv..

[55] Friedrich T., Durante M., Scholz M. (2012). Modeling cell survival after photon irradiation based on double-strand break clustering in megabase pair chromatin loops. Radiat. Res..

[56] Friedrich T., Scholz U., Elsässer T., Durante M., Scholz M. (2013). Systematic analysis of RBE and related quantities using a database of cell survival experiments with ion beam irradiation. J. Radiat. Res..

[57] Herr L., Friedrich T., Durante M., Scholz M. (2014). A model of photon cell killing based on the spatio-temporal clustering of DNA damage in higher order chromatin structures. PLoS ONE.

[58] Erfle H., Neumann B., Liebel U., Rogers P., Held M., Walter T., Ellenberg J., Pepperkok R. (2007). Reverse transfection on cell arrays for high content screening microscopy. Nat. Protoc..

[59] Pontén J., Saksela E. (1967). Two established in vitro cell lines from human mesenchymal tumours. Int. J. Cancer.

[60] Chagin V.O., Casas-Delucchi C.S., Reinhart M., Schermelleh L., Markaki Y., Maiser A., Bolius J.J., Bensimon A., Fillies M., Domaing P. (2016). 4D Visualization of replication foci in mammalian cells corresponding to individual replicons. Nat. Commun..

[61] DepMap Data Explorer. https://depmap.org/portal/interactive/?yDataset=&yFeature=&x=slice%2Fcopy_number_absolute%2F5524%2Fentity_id.

[62] Subcellular-CTCF-The Human Protein Atlas. https://www.proteinatlas.org/ENSG00000102974-CTCF/subcellular.

[63] Guénet J.L. (2005). The mouse genome. Genome Res..

[64] Hufnagl A., Herr L., Friedrich T., Durante M., Taucher-Scholz G., Scholz M. (2015). The link between cell-cycle dependent radiosensitivity and repair pathways: A model based on the local, sister-chromatid conformation dependent switch between NHEJ and HR. DNA Repair.

[65] Hooper M., Hardy K., Handyside A., Hunter S., Monk M. (1987). HPRT-deficient (Lesch-Nyhan) mouse embryos derived from germline colonization by cultured cells. Nature.

[66] Pękowska A., Klaus B., Xiang W., Severino J., Daigle N., Klein F.A., Oleś M., Casellas R., Ellenberg J., Steinmetz L.M. (2018). Gain of CTCF-Anchored Chromatin Loops Marks the Exit from Naive Pluripotency. Cell Syst..

[67] Johnston P.J., Olive P.L., Bryant P.E. (1997). Higher-order chromatin structure-dependent repair of DNA double-strand breaks: Modeling the elution of DNA from nucleoids. Radiat. Res..

[68] Tommasino F., Friedrich T., Scholz U., Taucher-Scholz G., Durante M., Scholz M. (2013). A DNA double-strand break kinetic rejoining model based on the local effect model. Radiat. Res..

[69] Tommasino F., Friedrich T., Scholz U., Taucher-Scholz G., Durante M., Scholz M. (2015). Application of the local effect model to predict DNA double-strand break rejoining after photon and high-LET irradiation. Radiat. Prot. Dosim..

[70] Tommasino F., Friedrich T., Jakob B., Meyer B., Durante M., Scholz M. (2015). Induction and Processing of the Radiation-Induced Gamma-H2AX Signal and Its Link to the Underlying Pattern of DSB: A Combined Experimental and Modelling Study. PLoS ONE.

[71] Ricci M.A., Manzo C., García-Parajo M.F., Lakadamyali M., Cosma M.P. (2015). Chromatin fibers are formed by heterogeneous groups of nucleosomes in vivo. Cell.

[72] Efroni S., Duttagupta R., Cheng J., Dehghani H., Hoeppner D.J., Dash C., Bazett-Jones D.P., Le Grice S., McKay R.D.G., Buetow K.H. (2008). Global transcription in pluripotent embryonic stem cells. Cell Stem Cell.

[73] Marks H., Kalkan T., Menafra R., Denissov S., Jones K., Hofemeister H., Nichols J., Kranz A., Stewart A.F., Smith A. (2012). The transcriptional and epigenomic foundations of ground state pluripotency. Cell.

[74] Banáth J.P., Bañuelos C.A., Klokov D., MacPhail S.M., Lansdorp P.M., Olive P.L. (2009). Explanation for excessive DNA single-strand breaks and endogenous repair foci in pluripotent mouse embryonic stem cells. Exp. Cell Res..

[75] Saretzki G., Armstrong L., Leake A., Lako M., von Zglinicki T. (2004). Stress defense in murine embryonic stem cells is superior to that of various differentiated murine cells. Stem Cells.

[76] Aladjem M.I., Spike B.T., Rodewald L.W., Hope T.J., Klemm M., Jaenisch R., Wahl G.M. (1998). ES cells do not activate p53-dependent stress responses and undergo p53-independent apoptosis in response to DNA damage. Curr. Biol..

[77] Cervantes R.B., Stringer J.R., Shao C., Tischfield J.A., Stambrook P.J. (2002). Embryonic stem cells and somatic cells differ in mutation frequency and type. Proc. Natl. Acad. Sci. USA.

[78] Lin T., Chao C., Saito S., Mazur S.J., Murphy M.E., Appella E., Xu Y. (2005). p53 induces differentiation of mouse embryonic stem cells by suppressing Nanog expression. Nat. Cell Biol..

[79] Tichy E.D., Stambrook P.J. (2008). DNA repair in murine embryonic stem cells and differentiated cells. Exp. Cell Res..

[80] Valerie K., Povirk L.F. (2003). Regulation and mechanisms of mammalian double-strand break repair. Oncogene.

[81] Sedelnikova O.A., Pilch D.R., Redon C., Bonner W.M. (2003). Histone H2AX in DNA damage and repair. Cancer Biol. Ther..

[82] Karagiannis T.C., El-Osta A. (2004). Double-strand breaks: Signaling pathways and repair mechanisms. Cell. Mol. Life Sci..

[83] Takata M., Sasaki M.S., Sonoda E., Morrison C., Hashimoto M., Utsumi H., Yamaguchi-Iwai Y., Shinohara A., Takeda S. (1998). Homologous recombination and non-homologous end-joining pathways of DNA double-strand break repair have overlapping roles in the maintenance of chromosomal integrity in vertebrate cells. EMBO J..

[84] Wang H., Zeng Z.C., Bui T.A., Sonoda E., Takata M., Takeda S., Iliakis G. (2001). Efficient rejoining of radiation-induced DNA double-strand breaks in vertebrate cells deficient in genes of the RAD52 epistasis group. Oncogene.

[85] Rothkamm K., Krüger I., Thompson L.H., Löbrich M. (2003). Pathways of DNA double-strand break repair during the mammalian cell cycle. Mol. Cell. Biol..

[86] Savatier P., Lapillonne H., Jirmanova L., Vitelli L., Samarut J. (2002). Analysis of the cell cycle in mouse embryonic stem cells. Methods Mol. Biol..

[87] Friedberg E.C., Meira L.B. (2006). Database of mouse strains carrying targeted mutations in genes affecting biological responses to DNA damage Version 7. DNA Repair.

[88] Yang Y.-G., Cortes U., Patnaik S., Jasin M., Wang Z.-Q. (2004). Ablation of PARP-1 does not interfere with the repair of DNA double-strand breaks, but compromises the reactivation of stalled replication forks. Oncogene.

[89] Francis R., Richardson C. (2007). Multipotent hematopoietic cells susceptible to alternative double-strand break repair pathways that promote genome rearrangements. Genes Dev..

[90] MacPhail S.H., Banáth J.P., Yu T.Y., Chu E.H.M., Lambur H., Olive P.L. (2003). Expression of phosphorylated histone H2AX in cultured cell lines following exposure to X-rays. Int. J. Radiat. Biol..

[91] Banáth J.P., Macphail S.H., Olive P.L. (2004). Radiation sensitivity, H2AX phosphorylation, and kinetics of repair of DNA strand breaks in irradiated cervical cancer cell lines. Cancer Res..

[92] Wada S., Van Khoa T., Kobayashi Y., Funayama T., Ogihara K., Ueno S., Ito N. (2005). Prediction of cellular radiosensitivity from DNA damage induced by gamma-rays and carbon ion irradiation in canine tumor cells. J. Vet. Med. Sci..

[93] Mirzayans R., Severin D., Murray D. (2006). Relationship between DNA double-strand break rejoining and cell survival after exposure to ionizing radiation in human fibroblast strains with differing ATM/p53 status: Implications for evaluation of clinical radiosensitivity. Int. J. Radiat. Oncol. Biol. Phys..

[94] Ward J.F. (1994). The complexity of DNA damage: Relevance to biological consequences. Int. J. Radiat. Biol..

[95] Ottolenghi A., Merzagora M., Tallone L., Durante M., Paretzke H.G., Wilson W.E. (1995). The quality of DNA double-strand breaks: A Monte Carlo simulation of the end-structure of strand breaks produced by protons and alpha particles. Radiat. Environ. Biophys..

[96] Sutherland B.M., Bennett P.V., Sidorkina O., Laval J. (2000). Clustered DNA damages induced in isolated DNA and in human cells by low doses of ionizing radiation. Proc. Natl. Acad. Sci. USA.

[97] Nikjoo H., O’Neill P., Wilson W.E., Goodhead D.T. (2001). Computational approach for determining the spectrum of DNA damage induced by ionizing radiation. Radiat. Res..

[98] Johnston P.J., MacPhail S.H., Banáth J.P., Olive P.L. (1998). Higher-order chromatin structure-dependent repair of DNA double-strand breaks: Factors affecting elution of DNA from nucleoids. Radiat. Res..

[99] Johnston P.J., MacPhail S.H., Stamato T.D., Kirchgessner C.U., Olive P.L. (1998). Higher-order chromatin structure-dependent repair of DNA double-strand breaks: Involvement of the V(D)J recombination double-strand break repair pathway. Radiat. Res..

[100] Friedland W., Jacob P., Paretzke H.G., Ottolenghi A., Ballarini F., Liotta M. (2006). Simulation of light ion induced DNA damage patterns. Radiat. Prot. Dosim..

[101] Stenerlöw B., Karlsson K.H., Cooper B., Rydberg B. (2003). Measurement of prompt DNA double-strand breaks in mammalian cells without including heat-labile sites: Results for cells deficient in nonhomologous end joining. Radiat. Res..

[102] Singh S.K., Wang M., Staudt C., Iliakis G. (2011). Post-irradiation chemical processing of DNA damage generates double-strand breaks in cells already engaged in repair. Nucleic Acids Res..

[103] Singh S.K., Bencsik-Theilen A., Mladenov E., Jakob B., Taucher-Scholz G., Iliakis G. (2013). Reduced contribution of thermally labile sugar lesions to DNA double strand break formation after exposure to heavy ions. Radiat. Oncol..

[104] Georgakilas A.G., O’Neill P., Stewart R.D. (2013). Induction and repair of clustered DNA lesions: What do we know so far?. Radiat. Res..

[105] gd Online Documentation. http://bio.gsi.de/DOCS/gd.html.

