# Supplementary material for: The Chromatin Architectural Protein CTCF Is Critical for Cell Survival upon Irradiation-Induced DNA Damage
Source: Int J Mol Sci. 2022 Mar 31;23(7):3896. doi: 10.3390/ijms23073896 (PMC8999573; doi:10.3390/ijms23073896)
Supplement: Supplementary file 1 [file ijms-23-03896-s001.zip › IJMS_Mamberti_et_al_supplementary_clean_version.pdf]

International Journal of
Molecular Sciences

MDPI

The chromatin architectural protein CTCF is critical for cell survival upon irradiation-induced DNA damage

Stefania Mamberti [1], Maruthi K. Pabba [1], Alexander Rapp [1], M. Cristina Cardoso [1,*] and Michael Scholz [2,*]

[1] Cell Biology and Epigenetics, Department of Biology, Technical University of Darmstadt, 64287 Darmstadt, Germany; ste.mamberti@gmail.com (S.M.); pabba.maruthi123@gmail.com (M.K.P.); rapp@bio.tu-darmstadt.de (A.R.)
[2] GSI Helmholtzzentrum für Schwerionenforschung GmbH, Biophysics division, 64291 Darmstadt, Germany
* Correspondence: cardoso@bio.tu-darmstadt.de (M.C.C.); m.scholz@gsi.de (M.S.); Tel: +49 6151 16 21882 (M.C.C.); Tel: +49 6159 711340 (M.S.)

SUPPLEMENTARY TABLES, FIGURES AND TEXT

List of contents:

Supplementary Figure S1: Image analysis pipeline for high-content wide-field images.

Supplementary Figure S2: Western blot analysis.

Supplementary Table S1: Cell line characteristics.

Supplementary Table S2: Primary and secondary antibody characteristics.

Supplementary Table S3: Imaging system characteristics.

Supplementary Table S4: Plot statistics.

Supplementary Figure S3: Boxplot data visualization.

Supplementary Table S5: esiRNA characteristics.

Supplementary Table S6: Survival data.

Supplementary Figure S4: Colony formation images.

Supplementary Figure S5: Survival curves and modeling predictions with equal y axis.

Supplementary Figure S6: Time course validation of the CTCF-degron kinetics.

Supplementary Figure S7: Screenshot of model implementation as Excel sheet.

Supplementary Figure S1: Image analysis pipeline for high-content wide-field images.

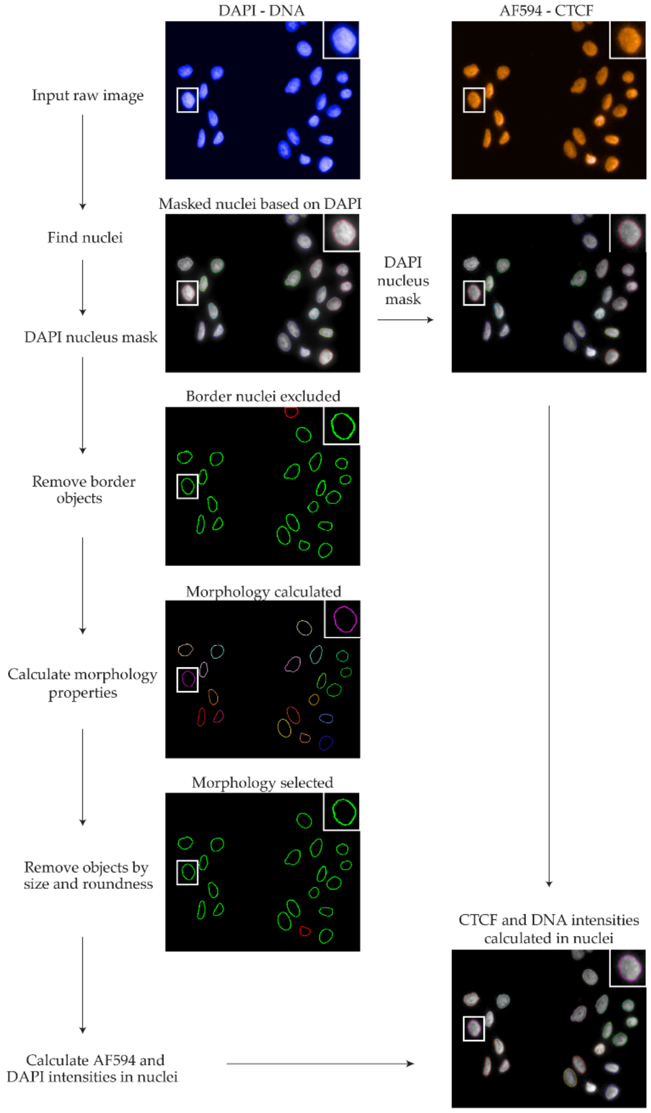

Calculations applied to the measured DNA and CTCF nuclear intensities	
Background subtraction	CTCF intensity sample - median (intensity sample without CTCF detection)
Normalization to DNA content	Background subtracted CTCF intensity sample / DAPI intensity sample
Normalization to control	CTCF intensity sample / median (CTCF intensity control sample)

Input raw images obtained with a high-content wide-field microscope (Supplementary Table S3) were analyzed to obtain nuclear intensity values of DNA (DAPI) and CTCF (A594 in HeLa Kyoto and U2OS cells; GFP in mESC-AID-CTCF cells). An example for HeLa Kyoto cells is shown. First, nuclei were identified and segmented based on the DNA signal. The nuclei touching the edge of the image were excluded. The morphological properties of nuclei were calculated and used to select nuclei. The nuclear DNA and CTCF intensities were calculated for the selected nuclei and plotted upon different calculations shown in the above table. Background subtraction was applied in Figure 1, (c, e), Figure 7 (b, c), Supplementary Figure S6 (b); for HeLa Kyoto and U2OS, the background was measured in cells stained with the primary antibody being omitted; for mESC-AID-CTCF cells, the background was measured in ES-E14TG2a WT cells which do not have any CTCF-GFP-tag. Normalization to DNA content was applied in Figure 1, (e) upon background subtraction. Normalization to control was applied in Figure 2 (b, c), Figure 7 (b, c), Supplementary Figure S6 (b).

Supplementary Figure S2: Western blot analysis.

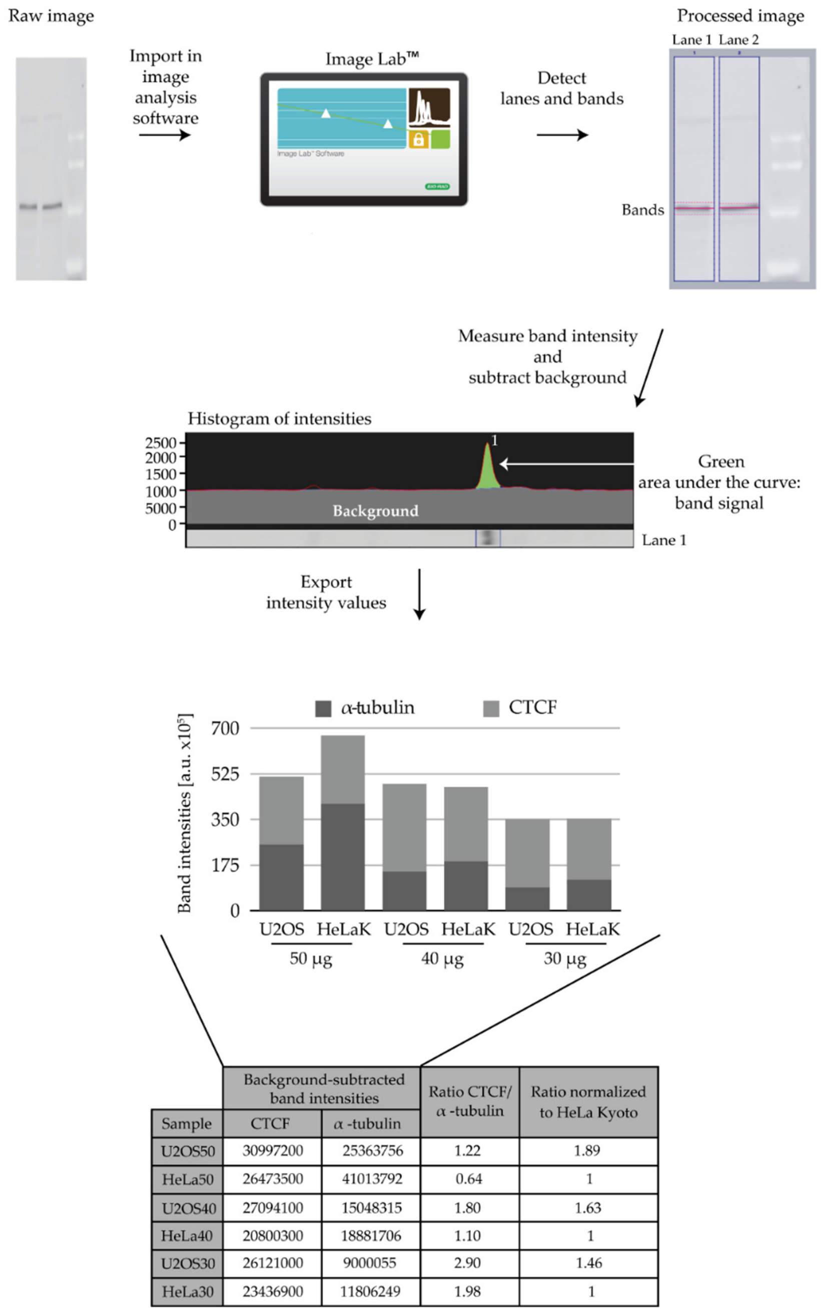

	Background-subtracted band intensities		Ratio CTCF/ α -tubulin	Ratio normalized to HeLa Kyoto
Sample	CTCF	α -tubulin		
U2OS50	30997200	25363756	1.22	1.89
HeLa50	26473500	41013792	0.64	1
U2OS40	27094100	15048315	1.80	1.63
HeLa40	20800300	18881706	1.10	1
U2OS30	26121000	9000055	2.90	1.46
HeLa30	23436900	11806249	1.98	1

The input raw image of the western blot was analyzed to calculate the intensity of the fluorescent bands detected with antibodies specific for CTCF and α-tubulin. The background from the untargeted membrane was subtracted to the absolute band intensities and the background-subtracted values of each lane normalized to the respective α-tubulin one. The resulting ratio for each cell line was normalized to the ratio obtained for HeLa Kyoto of the corresponding loaded protein amount (e.g. U2OS50 CTCF/α-tubulin ratio divided by HeLa50 CTCF/α-tubulin ratio). The three U2OS/HeLa Kyoto ratios were then averaged to obtain the "Mean" value shown in Figure 1 (h). For simplicity, in this figure HeLa Kyoto is abbreviated HeLaK or HeLa and the name of each sample in the table contains the loaded protein amount (e.g. U2OS50 = 50 μg of U2OS whole lysate).

Supplementary Table S1: Cell line characteristics.

Name	Species	Type	Identifier	Reference
HeLa Kyoto	*Homo sapiens*	cervical carcinoma	RRID*: CVCL_1922	[1]
U2OS	*Homo sapiens*	osteosarcoma	RRID: CVCL_0042	[2]
mESC-AID-CTCF	*Mus musculus*	embryonic stem	EN52.9.1	[3]
ES-E14TG2a	*Mus musculus*	embryonic stem	RRID: CVCL_9108	[4]

***RRID: Resource Identification Initiative Identifier**

Supplementary Table S2: Primary and secondary antibody characteristics.

Reactivity (clone)	Host	Dilution	Application	Cat #	Company/Reference
anti-CTCF	rabbit	1:800, 1:1000	IF*, WB**	2899	Cell Signaling Technology, Danvers, Massachusetts, USA
anti-α-tubulin (DM1A)	mouse	1:5000	WB	T9026	Sigma Aldrich, Merck KGaA, Darmstadt, Deutschland
anti-rabbit IgG AlexaFluor 594	goat	1:800	IF	111-585-144	Jackson ImmunoResearch Europe Ltd, Cambridge House, UK
anti-rabbit IgG AlexaFluor 488	goat	1:1000	WB	A11034	Invitrogen, Thermo Fisher, Waltham, Massachusetts, USA
anti-mouse IgG Cy5	donkey	1:1000	WB	715-175-150	Jackson ImmunoResearch Europe Ltd, Cambridge House, UK

*** IF: immunofluorescence; ** WB: western blot.**

Supplementary Table S3: Imaging system characteristics.

Microscope/ Company	Lasers/lamps	Filters (ex. & em. [nm]) *	Objectives/ lenses	Detection system	Application
Operetta® high content screening microscopy/ PerkinElmer Life Sciences, UK	Xenon fiber-optic light source, 300 W, 360 – 640 nm continuous spectrum	405/488/561** 405: ex.* 360-400 & em.* 410-480 488: ex. 460-490 & em. 500-550 561: ex. 560-580 & em. 590-640	40x air (0.95 NA) long WD***	14-bit Jenoptik CMOS	high content screening microscopy
Amersham™ Imager 600/GE Healthcare, Chicago, USA	Fluorescence epi illumination	488 BP40: ex. 520 & em. 505 Cy5 BP40: ex. 630 & em. 705	large aperture f/0.85 FUJINON™	16-bit Peltier cooled Fujifilm Super CCD	western blot detection

***ex.: excitation & em.: emission, ** dichroic specification, *** WD: working distance.**

Supplementary Table S4: Plot statistics.

Figure	Cell line	# biological replicate / Sample	n*	Median	Mean	StDev**
**1C**	**HK**	CTCF-stained	10535	211984	218612.2	71008.61
	**U2OS**	CTCF-stained	6189	433944	472436.1	224604.9
		Secondary Ab	23062	42593	44628.13	14203.24
**1E**	**HK**	CTCF-stained	10535	0.124	0.127	0.03
	**U2OS**	CTCF-stained	6189	0.213	0.220	0.09
**2B**	**HK**	#1 GFP KD	16371	119417	125964.87	48122.56
		#1 GFP KD norm.	16371	1	1.055	0.40
		#1 CTCF KD – 24 h	25001	74500	8018.61	34354
		#1 CTCF KD – 24 h norm.	25001	0.624	0.671	0.29
		#1 CTCF KD – 48 h	5256	106585	113491.49	46631.52
		#1 CTCF KD – 48 h norm.	5256	0.893	0.950	0.39
		#1 CTCF KD – 72 h	10830	94238.5	96642.94	28445.63
		#1 CTCF KD – 72 h norm.	10830	0.789	0.809	0.24
		#2 GFP KD	9006	187191.5	214236.6	103824.3
		#2 GFP KD norm.	9006	1	1.144	0.55
		#2 CTCF KD – 24 h	1984	114575	120246.6	39725.25
		#2 CTCF KD – 24 h norm.	1984	0.612	0.642	0.21
		#2 CTCF KD – 48 h	5325	114618	120878.1	44490.31
		#2 CTCF KD – 48 h norm.	5325	0.612	0.646	0.24
		#2 CTCF KD – 72 h	5539	122086	125749.2	40343.11
		#2 CTCF KD – 72 h norm.	5539	0.652	0.672	0.22
		#3 GFP KD	25852	186725	197557.8	78690.65
		#3 GFP KD norm.	25852	1	1.058	0.42
		#3 CTCF KD – 24 h	4714	118828	123747	39584.56
		#3 CTCF KD – 24 h norm.	4714	0.636	0.663	0.21
		#3 CTCF KD – 48 h	4934	107419	112492.3	38473.35
		#3 CTCF KD – 48 h norm.	4934	0.575	0.602	0.21
		#3 CTCF KD – 72 h	8750	114225	118252.1	37748.7
		#3 CTCF KD – 72 h norm.	8750	0.612	0.633	0.20

		GFP KD merged	51229	1	1.072	0.44
		CTCF KD – 24 h merged	31699	0.625	0.668	0.27
		CTCF KD – 48 h merged	15515	0.665	0.735	0.33
		CTCF KD – 72 h merged	25119	0.693	0.718	0.24
**2C**	**U2OS**	#1 GFP KD<br>#1 GFP KD norm.	2648<br>2648	584249.5<br>1	678319.04<br>1.161	351112.56<br>0.60
		#1 CTCF KD – 24 h<br>#1 CTCF KD – 24 h norm.	1640<br>1640	298600<br>0.511	313054.54<br>0.536	87753.73<br>0.15
		#1 CTCF KD – 48 h<br>#1 CTCF KD – 48 h norm.	2424<br>2424	288488<br>0.494	301797.55<br>0.517	89412.46<br>0.15
		#1 CTCF KD – 72 h<br>#1 CTCF KD – 72 h norm.	1174<br>1174	608136.5<br>1.041	638074.45<br>1.092	260539.93<br>0.45
		#2 GFP KD<br>#2 GFP KD norm.	1315<br>1315	954110<br>1	1024028<br>1.073	362094.9<br>0.38
		#2 CTCF KD – 24 h<br>#2 CTCF – 24 h norm.	1040<br>1040	480750<br>0.504	536206.3<br>0.562	249541.6<br>0.26
		#2 CTCF KD – 48 h<br>#2 CTCF KD – 48 h norm.	602<br>602	534528<br>0.560	601806<br>0.631	251735.9<br>0.26
		#2 CTCF KD – 72 h<br>#2 CTCF KD – 72 h norm.	471<br>471	462839<br>0.485	478718.1<br>0.502	186801.3<br>0.20
		#3 GFP KD<br>#3 GFP KD norm.	317<br>317	1812784<br>1	1864035<br>1.028	626358.7<br>0.35
		#3 CTCF KD – 24 h<br>#3 CTCF KD – 24 h norm.	324<br>324	937947.5<br>0.517	985970<br>0.544	314408.3<br>0.17
		#3 CTCF KD – 48 h<br>#3 CTCF KD – 48 h norm.	259<br>259	1155118<br>0.637	1220088<br>0.673	467461.1<br>0.26
		#3 CTCF KD – 72 h<br># CTCF KD – 72 h norm.	288<br>288	1128024<br>0.622	1205586<br>0.665	470095.8<br>0.26
		GFP KD merged	4280	1	1.124	0.53
		CTCF KD – 24 h merged	3004	0.510	0.546	0.20
		CTCF KD – 48 h merged	3285	0.514	0.550	0.20
		CTCF KD – 72 merged	1933	0.789	0.885	0.46

**7B**	**AID**	#1 – 0 µM ^{AUX}	2154	57.56	58.89	12.67
		#2 – 0 µM ^{AUX}	4292	59.69	60.00	10.85
		#3 – 0 µM ^{AUX}	1952	57.77	58.05	11.54
		#4 – 0 µM ^{AUX}	3680	58.72	58.82	11.16
		0 µM $^{\text{AUX merged}}$	12078	58.81	59.13	11.42
		0 µM $^{\text{AUX subtr.}}$	12078	41.86	42.18	11.42
		0 µM $^{\text{AUX norm.}}$	12078	1	1.007	0.27
		#1 – 25 µM ^{AUX}	3605	18.24	21.28	10.06
		#2 – 25 µM ^{AUX}	2284	18.16	20.27	7.55
		#3 – 25 µM ^{AUX}	5540	19.02	20.77	6.91
		#4 – 25 µM ^{AUX}	6274	18.55	20.00	6.17
		25 µM $^{\text{AUX merged}}$	17703	18.58	20.54	7.53
		25 µM $^{\text{AUX subtr.}}$	17703	1.63	3.59	7.53
		25 µM $^{\text{AUX norm.}}$	17703	0.039	0.086	0.18
		#1 – 500 µM ^{AUX}	2215	17.66	19.13	6.00
		#2 – 500 µM ^{AUX}	3163	16.38	17.96	6.66
		#3 – 500 µM ^{AUX}	4349	16.22	19.37	10.37
		#4 – 500 µM ^{AUX}	7708	15.68	16.68	4.90
		500 µM $^{\text{AUX merged}}$	17435	16.17	17.90	7.17
		500 µM $^{\text{AUX subtr.}}$	17435	-0.78	0.95	7.17
		500 µM $^{\text{AUX norm.}}$	17435	-0.019	0.023	0.17
		#1 – 1000 µM ^{AUX}	2082	15.56	17.19	6.83
		#2 – 1000 µM ^{AUX}	3129	16.28	17.63	6.55
		#3 – 1000 µM ^{AUX}	1535	17.04	18.14	5.37
		#4 – 1000 µM ^{AUX}	4162	16.57	17.66	5.04
		1000 µM $^{\text{AUX merged}}$	10908	16.31	17.63	5.92
		1000 µM $^{\text{AUX subtr.}}$	10908	-0.64	0.68	5.92
		1000 µM $^{\text{AUX norm.}}$	10908	-0.015	0.016	0.14
	**ES14**	WT untagged	47399	16.95	18.35	5.46
**SF5**	**AID**	#1 – 25 µM – 0 h ^{AUX}	3480	49.53	50.03	9.05
		#1 – 25 µM – 0.5 h ^{AUX}	1763	64.69	65.08	13.12
		#1 – 25 µM – 1 h ^{AUX}	1944	58.29	58.42	13.45
		#1 – 25 µM – 2 h ^{AUX}	2017	34.05	34.96	12.67
		#1 – 25 µM – 3 h ^{AUX}	2910	28.04	30.54	11.27
		#1 – 25 µM – 4 h ^{AUX}	4086	22.23	25.83	9.46
		#1 – 25 µM – 0.5 h ^{WA}	3411	20.53	23.25	7.62
		#1 – 25 µM – 1 h ^{WA}	4325	25.83	29.51	10.25
		#1 – 25 µM – 2 h ^{WA}	2981	26.75	29.62	8.98
		#1 – 25 µM – 4 h ^{WA}	7432	28.37	29.98	6.89
		#1 – 25 µM – 6 h ^{WA}	2725	41.23	42.83	9.84
		#1 – 25 µM – 8 h ^{WA}	4082	36.71	37.28	7.15
		#1 – 25 µM – 18 h ^{WA}	6337	76.32	76.21	14.50
		#1 – 25 µM – 24 h ^{WA}	11137	58.32	61.36	14.15
		#2 – 25 µM – 0 h ^{AUX}	2622	54.02	54.33	8.64
		#2 – 25 µM – 0.5 h ^{AUX}	2171	59.39	59.09	11.77

		#2 – 25 µM – 1 h ^{AUX}	3918	51.00	51.24	9.45
		#2 – 25 µM – 2 h ^{AUX}	5749	44.53	44.69	15.06
		#2 – 25 µM – 3 h ^{AUX}	2915	28.92	31.13	11.51
		#2 – 25 µM – 4 h ^{AUX}	2305	22.39	27.00	11.11
		#2 – 25 µM – 0.5 h ^{WA}	2977	22.53	27.03	10.75
		#2 – 25 µM – 1 h ^{WA}	2341	21.74	25.80	9.74
		#2 – 25 µM – 2 h ^{WA}	4189	24.66	28.20	9.77
		#2 – 25 µM – 4 h ^{WA}	4935	34.32	36.28	8.99
		#2 – 25 µM – 6 h ^{WA}	3822	40.06	41.74	9.79
		#2 – 25 µM – 8 h ^{WA}	3810	41.11	42.63	9.51
		#2 – 25 µM – 24 h ^{WA}	9749	75.08	75.00	13.97
		#3 – 25 µM – 0 h ^{AUX}	812	43.31	43.48	6.65
		#3 – 25 µM – 0.5 h ^{AUX}	1715	60.53	61.02	10.47
		#3 – 25 µM – 1 h ^{AUX}	3915	51.99	52.21	10.14
		#3 – 25 µM – 2 h ^{AUX}	6539	32.95	33.20	10.32
		#3 – 25 µM – 3 h ^{AUX}	4591	29.23	32.19	12.69
		#3 – 25 µM – 4 h ^{AUX}	3153	20.29	23.18	7.85
		#3 – 25 µM – 0.5 h ^{WA}	3054	21.17	23.73	7.04
		#3 – 25 µM – 1 h ^{WA}	2072	24.52	27.75	10.31
		#3 – 25 µM – 2 h ^{WA}	3046	42.70	45.09	12.88
		#3 – 25 µM – 4 h ^{WA}	846	41.35	46.53	20.77
		#3 – 25 µM – 6 h ^{WA}	769	56.41	59.07	16.39
		#3 – 25 µM – 8 h ^{WA}	1404	51.30	53.55	18.11
		#3 – 25 µM – 18 h ^{WA}	5461	83.89	83.08	14.49
		#3 – 25 µM – 24 h ^{WA}	3929	67.43	68.02	14.81
		25 µM – 0 h $^{\mathrm{AUX\ merged}}$	6914	50.29	50.89	9.27
		25 µM – 0.5 h $^{\mathrm{AUX\ merged}}$	5649	61.16	61.54	12.11
		25 µM – 1 h $^{\mathrm{AUX\ merged}}$	9777	52.5	53.06	10.96
		25 µM – 2 h $^{\mathrm{AUX\ merged}}$	14305	36.78	38.06	13.87
		25 µM – 3 h $^{\mathrm{AUX\ merged}}$	10416	28.80	31.43	12.00
		25 µM – 4 h $^{\mathrm{AUX\ merged}}$	9544	21.64	25.24	9.53
		25 µM – 0.5 h $^{\mathrm{WA\ merged}}$	9442	21.42	24.60	8.73
		25 µM – 1 h $^{\mathrm{WA\ merged}}$	8738	24.61	28.10	10.25
		25 µM – 2 h $^{\mathrm{WA\ merged}}$	10216	30.43	33.65	12.97
		25 µM – 4 h $^{\mathrm{WA\ merged}}$	13213	30.87	33.39	10.26
		25 µM – 6 h $^{\mathrm{WA\ merged}}$	7316	41.51	43.97	11.89
		25 µM – 8 h $^{\mathrm{WA\ merged}}$	9296	39.46	41.93	11.79
		25 µM – 18 h $^{\mathrm{WA\ merged}}$	11798	79.51	79.39	14.90
		25 µM – 24 h $^{\mathrm{WA\ merged}}$	24815	65.62	67.77	15.50
		25 µM – 0 h $^{\mathrm{AUX\ norm.}}$	6914	1	1.02	0.29
		25 µM – 0.5 h $^{\mathrm{AUX\ norm.}}$	5649	1.35	1.36	0.38
		25 µM – 1 h $^{\mathrm{AUX\ norm.}}$	9777	1.07	1.09	0.35
		25 µM – 2 h $^{\mathrm{AUX\ norm.}}$	14305	0.57	0.61	0.44
		25 µM – 3 h $^{\mathrm{AUX\ norm.}}$	10416	0.32	0.40	0.38
		25 µM – 4 h $^{\mathrm{AUX\ norm.}}$	9544	0.09	0.20	0.30
		25 µM – 0.5 h $^{\mathrm{WA\ norm.}}$	9442	0.08	0.18	0.28
		25 µM – 1 h $^{\mathrm{WA\ norm.}}$	8738	0.18	0.29	0.33
		25 µM – 2 h $^{\mathrm{WA\ norm.}}$	10216	0.37	0.47	0.41
		25 µM – 4 h $^{\mathrm{WA\ norm.}}$	13213	0.38	0.46	0.33

		25 µM – 6 h $^{\text{WA norm.}}$	7316	0.72	0.80	0.38
		25 µM – 8 h $^{\text{WA norm.}}$	9296	0.66	0.73	0.37
		25 µM – 18 h $^{\text{WA norm.}}$	11798	1.93	1.92	0.47
		25 µM – 24 h $^{\text{WA norm.}}$	24815	1.49	1.56	0.49
	**AID**	#1 – 500 µM – 0 h ^{AUX}	3236	54.88	55.18	10.43
		#1 – 500 µM – 0.5 h ^{AUX}	2996	54.77	55.74	9.32
		#1 – 500 µM – 1 h ^{AUX}	2842	46.58	46.02	12.25
		#1 – 500 µM – 2 h ^{AUX}	2982	21.09	25.95	11.69
		#1 – 500 µM – 3 h ^{AUX}	1640	17.92	24.51	12.61
		#1 – 500 µM – 4 h ^{AUX}	3600	16.73	19.52	7.69
		#1 – 500 µM – 0.5 h ^{WA}	2536	19.83	22.63	8.25
		#1 – 500 µM – 1 h ^{WA}	740	15.08	16.07	3.48
		#1 – 500 µM – 2 h ^{WA}	4373	20.89	22.99	7.52
		#1 – 500 µM – 4 h ^{WA}	2523	24.55	27.49	10.28
		#1 – 500 µM – 6 h ^{WA}	4198	29.00	32.74	12.38
		#1 – 500 µM – 8 h ^{WA}	4150	31.69	32.04	6.31
		#1 – 500 µM – 18 h ^{WA}	4931	44.62	44.95	8.26
		#1 – 500 µM – 24 h ^{WA}	8888	56.60	57.00	11.75
		#2 – 500 µM – 0 h ^{AUX}	2471	61.28	61.95	11.04
		#2 – 500 µM – 0.5 h ^{AUX}	1351	64.48	64.03	13.79
		#2 – 500 µM – 1 h ^{AUX}	3655	46.01	46.53	9.49
		#2 – 500 µM – 2 h ^{AUX}	3706	25.35	29.06	12.24
		#2 – 500 µM – 3 h ^{AUX}	4582	25.49	30.17	12.63
		#2 – 500 µM – 4 h ^{AUX}	4131	19.25	22.31	8.13
		#2 – 500 µM – 0.5 h ^{WA}	7104	22.60	25.83	9.26
		#2 – 500 µM – 1 h ^{WA}	2988	26.46	29.14	8.42
		#2 – 500 µM – 2 h ^{WA}	2938	23.01	25.15	7.13
		#2 – 500 µM – 4 h ^{WA}	3342	31.27	32.93	7.95
		#2 – 500 µM – 6 h ^{WA}	2163	29.54	30.67	7.07
		#2 – 500 µM – 8 h ^{WA}	4596	37.24	38.69	9.90
		#2 – 500 µM – 18 h ^{WA}	4765	67.91	68.95	16.30
		#2 – 500 µM – 24 h ^{WA}	5607	54.27	54.70	9.94
		#3 – 500 µM – 0 h ^{AUX}	2482	45.91	46.45	8.09
		#3 – 500 µM – 0.5 h ^{AUX}	2156	56.63	56.52	10.58
		#3 – 500 µM – 1 h ^{AUX}	3006	50.13	49.93	10.41
		#3 – 500 µM – 2 h ^{AUX}	3253	33.04	35.18	13.41
		#3 – 500 µM – 3 h ^{AUX}	4506	25.79	29.54	10.20
		#3 – 500 µM – 4 h ^{AUX}	3613	24.07	27.44	8.89
		#3 – 500 µM – 0.5 h ^{WA}	1311	21.28	24.37	8.55
		#3 – 500 µM – 1 h ^{WA}	2981	23.31	26.19	8.12
		#3 – 500 µM – 2 h ^{WA}	1690	22.33	24.43	7.06
		#3 – 500 µM – 4 h ^{WA}	2840	24.16	26.22	8.29
		#3 – 500 µM – 6 h ^{WA}	2738	32.16	33.32	7.72
		#3 – 500 µM – 8 h ^{WA}	3708	33.98	34.79	7.02
		#3 – 500 µM – 18 h ^{WA}	7116	56.93	58.05	11.92
		#3 – 500 µM – 24 h ^{WA}	6922	71.66	71.23	14.82
		500 µM – 0 h $^{\text{AUX merged}}$	8189	53.73	54.57	11.67

		500 μM – 0.5 h $^{\text{AUX merged}}$	6503	56.75	57.72	11.28
		500 μM – 1 h $^{\text{AUX merged}}$	9503	47.47	47.45	10.80
		500 μM – 2 h $^{\text{AUX merged}}$	9941	26.83	30.13	13.03
		500 μM – 3 h $^{\text{AUX merged}}$	10728	24.90	29.04	11.83
		500 μM – 4 h $^{\text{AUX merged}}$	11344	20.16	23.06	8.85
		500 μM – 0.5 h $^{\text{WA merged}}$	10951	21.83	24.92	9.05
		500 μM – 1 h $^{\text{WA merged}}$	6709	24.38	26.39	8.80
		500 μM – 2 h $^{\text{WA merged}}$	9001	21.74	23.96	7.38
		500 μM – 4 h $^{\text{WA merged}}$	8705	26.71	29.17	9.30
		500 μM – 6 h $^{\text{WA merged}}$	9099	30.22	32.42	10.08
		500 μM – 8 h $^{\text{WA merged}}$	12454	34.07	35.32	8.48
		500 μM – 18 h $^{\text{WA merged}}$	16812	55.06	57.30	15.45
		500 μM – 24 h $^{\text{WA merged}}$	21417	59.36	60.99	14.32
		500 μM – 0 h $^{\text{AUX norm.}}$	8189	1	1.02	0.33
		500 μM – 0.5 h $^{\text{AUX norm.}}$	6503	1.09	1.11	0.32
		500 μM – 1 h $^{\text{AUX norm.}}$	9503	0.82	0.82	0.31
		500 μM – 2 h $^{\text{AUX norm.}}$	9941	0.23	0. 32	0.37
		500 μM – 3 h $^{\text{AUX norm.}}$	10728	0.17	0.29	0.34
		500 μM – 4 h $^{\text{AUX norm.}}$	11344	0.04	0.12	0.25
		500 μM – 0.5 h $^{\text{WA norm.}}$	10951	0.09	0.17	0.26
		500 μM – 1 h $^{\text{WA norm.}}$	6709	0.16	0.22	0.25
		500 μM – 2 h $^{\text{WA norm.}}$	9001	0.08	0.15	0.21
		500 μM – 4 h $^{\text{WA norm.}}$	8705	0.23	0.30	0.27
		500 μM – 6 h $^{\text{WA norm.}}$	9099	0.33	0.39	0.29
		500 μM – 8 h $^{\text{WA norm.}}$	12454	0.44	0.47	0.24
		500 μM – 18 h $^{\text{WA norm.}}$	16812	1.04	1.10	0.44
		500 μM – 24 h $^{\text{WA norm.}}$	21417	1.16	1.21	0.41
	**AID**	#1 – 1000 μM – 0 h ^{AUX}	2026	59.79	60.26	10.73
		#1 – 1000 μM – 0.5 h ^{AUX}	1805	71.94	71.74	14.92
		#1 – 1000 μM – 1 h ^{AUX}	4211	52.24	53.49	18.07
		#1 – 1000 μM – 2 h ^{AUX}	2863	20.56	25.09	12.04
		#1 – 1000 μM – 3 h ^{AUX}	4205	22.00	24.76	10.83
		#1 – 1000 μM – 4 h ^{AUX}	5232	20.93	23.18	8.11
		#1 – 1000 μM – 0.5 h ^{WA}	4139	18.17	20.32	6.33
		#1 – 1000 μM – 1 h ^{WA}	4184	19.38	21.27	6.56
		#1 – 1000 μM – 2 h ^{WA}	1070	22.05	23.68	6.99
		#1 – 1000 μM – 4 h ^{WA}	2975	27.55	29.65	8.91
		#1 – 1000 μM – 6 h ^{WA}	5131	29.31	31.88	9.66
		#1 – 1000 μM – 8 h ^{WA}	3891	42.12	43.90	11.39
		#1 – 1000 μM – 18 h ^{WA}	6405	47.03	48.06	11.13
		#1 – 1000 μM – 24 h ^{WA}	10508	55.15	55.73	11.31
		#2 – 1000 μM – 0 h ^{AUX}	5519	50.28	50.88	7.95
		#2 – 1000 μM – 0.5 h ^{AUX}	3005	61.79	61.94	14.52
		#2 – 1000 μM – 1 h ^{AUX}	1321	61.29	61.88	17.06
		#2 – 1000 μM – 2 h ^{AUX}	2386	39.20	42.63	16.36
		#2 – 1000 μM – 3 h ^{AUX}	352	19.54	25.09	12.24
		#2 – 1000 μM – 4 h ^{AUX}	892	24.22	27.21	10.69
		#2 – 1000 μM – 0.5 h ^{WA}	961	23.92	27.77	11.74
		#2 – 1000 μM – 1 h ^{WA}	2264	30.39	33.54	12.72

		#2 – 1000 µM – 2 h ^{WA}	881	21.46	24.19	8.31
		#2 – 1000 µM – 4 h ^{WA}	997	31.56	34.49	11.75
		#2 – 1000 µM – 6 h ^{WA}	185	34.24	36.72	10.91
		#2 – 1000 µM – 8 h ^{WA}	1634	36.32	39.25	14.13
		#2 – 1000 µM – 18 h ^{WA}	1963	44.33	44.71	8.41
		#2 – 1000 µM – 24 h ^{WA}	6638	60.73	61.65	14.68
		#3 – 1000 µM – 0 h ^{AUX}	2779	40.53	40.96	6.60
		#3 – 1000 µM – 0.5 h ^{AUX}	3030	43.38	43.44	7.38
		#3 – 1000 µM – 1 h ^{AUX}	2254	34.64	34.82	8.78
		#3 – 1000 µM – 2 h ^{AUX}	4731	20.44	24.50	11.02
		#3 – 1000 µM – 3 h ^{AUX}	2707	18.41	23.83	12.28
		#3 – 1000 µM – 4 h ^{AUX}	10032	17.69	21.42	9.40
		#3 – 1000 µM – 0.5 h ^{WA}	3368	19.33	21.43	6.31
		#3 – 1000 µM – 1 h ^{WA}	2743	22.21	24.35	6.96
		#3 – 1000 µM – 2 h ^{WA}	1687	20.64	21.66	5.15
		#3 – 1000 µM – 4 h ^{WA}	3324	20.76	21.86	4.48
		#3 – 1000 µM – 6 h ^{WA}	2638	36.11	37.62	9.34
		#3 – 1000 µM – 18 h ^{WA}	6374	59.64	60.16	12.36
		#3 – 1000 µM – 24 h ^{WA}	12886	45.02	45.26	7.37
		1000 µM – 0 h $^{\text{AUX merged}}$	10324	49.22	50.05	10.54
		1000 µM – 0.5 h $^{\text{AUX merged}}$	7840	54.31	57.05	16.84
		1000 µM – 1 h $^{\text{AUX merged}}$	7786	46.52	49.51	18.58
		1000 µM – 2 h $^{\text{AUX merged}}$	9980	25.14	29.00	14.88
		1000 µM – 3 h $^{\text{AUX merged}}$	7264	20.60	24.43	11.47
		1000 µM – 4 h $^{\text{AUX merged}}$	16156	18.76	22.31	9.20
		1000 µM – 0.5 h $^{\text{WA merged}}$	8468	19.11	21.60	7.49
		1000 µM – 1 h $^{\text{WA merged}}$	9191	21.78	25.21	9.91
		1000 µM – 2 h $^{\text{WA merged}}$	3638	21.28	22.87	6.69
		1000 µM – 4 h $^{\text{WA merged}}$	7296	24.83	26.76	9.10
		1000 µM – 6 h $^{\text{WA merged}}$	7954	31.43	33.90	9.96
		1000 µM – 8 h $^{\text{WA merged}}$	5525	41.05	42.52	12.44
		1000 µM – 18 h $^{\text{WA merged}}$	14742	51.22	52.85	13.09
		1000 µM – 24 h $^{\text{WA merged}}$	30032	50.57	52.55	12.66
		1000 µM – 0 h $^{\text{AUX norm.}}$	10324	1	1.03	0.35
		1000 µM – 0.5 h $^{\text{AUX norm.}}$	7840	1.17	1.26	0.55
		1000 µM – 1 h $^{\text{AUX norm.}}$	7786	0.91	1.01	0.61
		1000 µM – 2 h $^{\text{AUX norm.}}$	9980	0.21	0.33	0.49
		1000 µM – 3 h $^{\text{AUX norm.}}$	7264	0.06	0.18	0.38
		1000 µM – 4 h $^{\text{AUX norm.}}$	16156	-0.002	0.11	0.30
		1000 µM – 0.5 h $^{\text{WA norm.}}$	8468	0.009	0.09	0.25
		1000 µM – 1 h $^{\text{WA norm.}}$	9191	0.10	0.21	0.33
		1000 µM – 2 h $^{\text{WA norm.}}$	3638	0.08	0.13	0.22
		1000 µM – 4 h $^{\text{WA norm.}}$	7296	0.20	0.26	0.30
		1000 µM – 6 h $^{\text{WA norm.}}$	7954	0.41	0.50	0.33
		1000 µM – 8 h $^{\text{WA norm.}}$	5525	0.73	0.78	0.41
		1000 µM – 18 h $^{\text{WA norm.}}$	14742	1.07	1.12	0.43
		1000 µM – 24 h $^{\text{WA norm.}}$	30032	1.04	1.11	0.42
	**ES14**	WT untagged	13829	18.82	22.12	11.42

***n: number of cells of all replicates (if not stated otherwise); **StDev: standard deviation;**
cell line name abbreviations: HK = HeLa Kyoto, AID = mESC-AID-CTCF, ES14 = ES-E14TG2a;
sample names: # = biological replicate number, norm. = normalized, merged = pooled replicates,
subtr. = background-subtracted, AUX = auxin, WA = auxin wash-off.

Supplementary Figure S3: Boxplot data visualization.

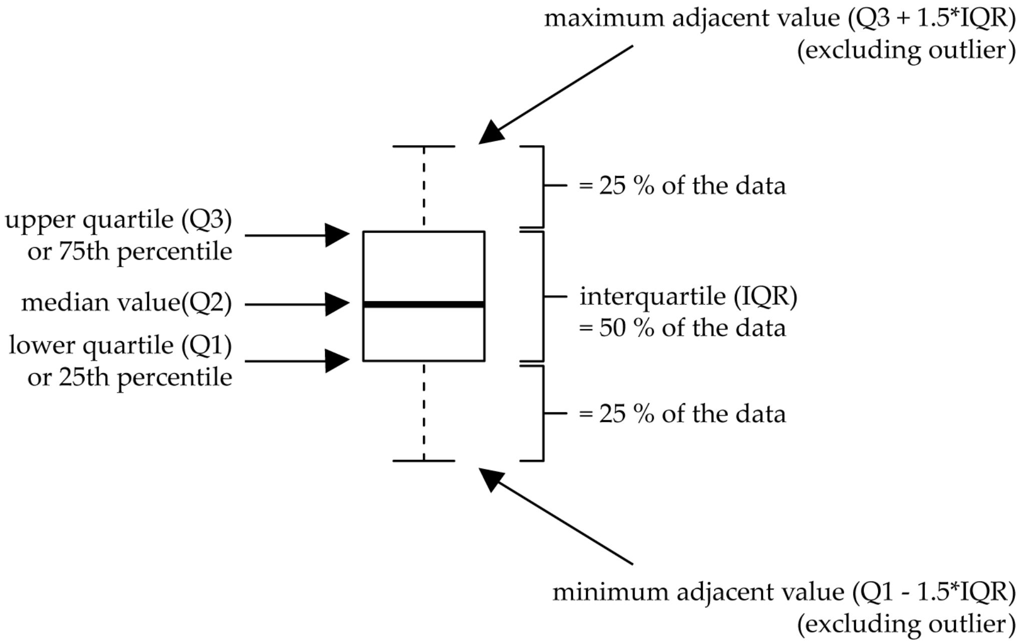

Boxplots allow to visualize the main statistics of a dataset. The "box" contains 50% of the observations, with the median value depicted as a line in it. The higher and lower borders of the box respectively represent the upper and lower quartile of the distribution. The remaining 50% of the observations is contained in the "whiskers" that depart from the box. The extreme values at the whiskers represent instead the maximum and minimum data points that are not considered outliers, as lying at +/- 1.5 times the interquartile range.

Supplementary Table S5: esiRNA characteristics.

Name	esiRNA cDNA target Sequence	Accession no.	Application	Reference
**EHU130111 Sigma-Aldrich MISSION® esiRNA targeting human CTCF**	AACAGCAGGAGGGTCTGCTATCAGAG<br>GTTAATGCAGAGAAAGTGGTTGGTAA<br>TATGAAGCCTCCAAAGCCAACAAAAA<br>TTAAAAAGAAAGGTGTAAAGAAGAC<br>ATTCCAGTGTGAGCTTTGCAGTTACAC<br>GTGTCCACGGCGTTCAAATTTGGATCG<br>TCACATGAAAAGCCACACTGATGAGA<br>GACCACACAAGTGCCATCTCTGTGGC<br>AGGGCATTCAGAACAGTCACCCTCCT<br>GAGGAATCACCTTAACACACACACAG<br>GTACTCGTCCTCACAAGTGCCCAGACT<br>GCGACATGGCCTTTGTGACCAGTGGA<br>GAATTGGTTCGGCATCGTCGTTACAAA<br>CACACCCACGAGAAGCCATTCAAGTG<br>TTCCATGTGCGATTACGCCAGTGTAGA<br>AGTCAGCAAATTAAAACGTCACATTC<br>GCTCTCATACTGGAGAGCGTCCGTTTC<br>AGTGCAGTTTGTGCAGTTATGCCAGCA<br>GGGACACATACAAGCTGAAAAGGCA<br>CATGA	Ensembl: ENSG00000102974<br><br>NCBI: NM_006565.3	CTCF mRNA silencing	https://www.sigmaaldrich.com/catalog/product/sigma/ehu130111?lang=de&region=DE
**EHUEGFP Sigma-Aldrich MISSION® esiRNA targeting EGFP**	GTGAGCAAGGGCGAGGAGCTGTTCAC<br>CGGGGTGGTGCCCATCCTGGTCGAGC<br>TGGACGGCGACGTAAACGGCCACAA<br>GTTCAGCGTGTCCGGCGAGGGCGAGG<br>GCGATGCCACCTACGGCAAGCTGACC<br>CTGAAGTTCATCTGCACCACCGGCAA<br>GCTGCCCGTGCCCTGGCCCACCCTCGT<br>GACCACCCTGACCTACGGCGTGCAGT<br>GCTTCAGCCGCTACCCCGACCACATG<br>AAGCAGCACGACTTCTTCAAGTCCGC<br>CATGCCCGAAGGCTACGTCCAGGAGC<br>GCACCATCTTCTTCAAGGACGACGGC<br>AACTACAAGACCCGCGCCGAGGTGAA<br>GTTCGAGGGCGACACCCTGGTGAACC<br>GCATCGAGCTGAAGGGCATCGACTTC<br>AAGGAGGACGGCAACATCCTGGGGC<br>ACAAGCTGGAGTACAACTACAACAGC<br>CACAACGTCTATATCATGGCCGACAA<br>GCAGAAGAACGGCATCAAGGTGAAC<br>TTCAAGATCCGCCACAACATCGAGGA<br>CGGCAGCGTGCAGCTCGCCGACCACT<br>ACCAGCAGAACACCCCCATCGGCGAC<br>GGCCCCGTGCTGCTGCCCGACAACCA<br>CTACCTGAGCACCCAGTCCGCCCTGA<br>GCAAAGACCCCAACGAGAAGCGCGA<br>TCACATGGTCCTGCTGGAGTTCGTGAC<br>CGCCGCCGGGATCACTCTCGGCATGG<br>ACGAGCTGTA	UniProtKB: C5MKY7<br><br>GenBank: MK387175.1	mock control for knockdown	https://www.sigmaaldrich.com/catalog/product/sigma/ehuegfp?lang=de&region=DE

Supplementary Table S6: Survival data.

Figure	Cell line	# biological replicate / Sample	X-rays dose (Gy)										
			0	1	2	3	4	5	6	7	8	9	10
3B	HK	#1 GFP KD raw*	551	349	304	270	251	210	181	124	80	50	18
			591	342	287	281	222	196	178	133	73	49	19
			475	-	-	-	-	200	160	106	74	53	27
		#1 GFP KD norm.**	1.02	0.65	0.56	0.50	0.47	0.39	0.34	0.23	0.15	0.09	0.03
			1.10	0.63	0.53	0.52	0.41	0.36	0.33	0.25	0.14	0.09	0.04
			0.88	-	-	-	-	0.37	0.30	0.20	0.14	0.10	0.05
		#1 GFP KD mean	1.00	0.64	0.55	0.51	0.44	0.37	0.32	0.22	0.14	0.09	0.04
		#1 GFP KD error***	0.04	0.03	0.03	0.03	0.03	0.03	0.02	0.02	0.02	0.01	0.01
		#1 CTCF KD raw	493	382	263	250	171	144	97	82	64	31	14
			500	386	268	253	167	152	100	80	70	37	10
			478	-	-	-	-	130	101	84	70	35	16
		#1 CTCF KD norm.	1.01	0.78	0.54	0.51	0.35	0.29	0.20	0.17	0.13	0.06	0.03
			1.02	0.79	0.55	0.52	0.34	0.31	0.20	0.16	0.14	0.08	0.02
			0.97	-	-	-	-	0.27	0.21	0.17	0.14	0.07	0.03
		#1 CTCF KD mean	1.00	0.78	0.54	0.51	0.34	0.29	0.20	0.17	0.14	0.07	0.03
		#1 CTCF KD error	0.05	0.04	0.03	0.03	0.03	0.02	0.02	0.02	0.02	0.01	0.01
		#2 GFP KD raw	633	469	440	386	265	235	183	135	103	52	29
			662	473	407	350	252	219	171	122	115	48	23
			637	480	432	313	240	208	174	129	97	58	21
		#2 GFP KD norm.	0.98	0.73	0.68	0.60	0.41	0.36	0.28	0.21	0.16	0.08	0.05
			1.03	0.73	0.63	0.54	0.39	0.34	0.27	0.19	0.18	0.07	0.04
			0.99	0.75	0.67	0.49	0.37	0.32	0.27	0.20	0.15	0.09	0.03
		#2 GFP KD mean	1.00	0.74	0.66	0.54	0.39	0.34	0.27	0.20	0.16	0.08	0.04
		#2 GFP KD error	0.04	0.03	0.03	0.03	0.02	0.02	0.02	0.02	0.02	0.01	0.01
		#2 CTCF KD raw	594	393	341	280	178	168	114	102	60	24	6
			604	367	312	248	180	162	120	90	54	30	9
			607	398	332	261	140	150	108	96	48	33	7
		#2 CTCF KD norm.	0.99	0.65	0.57	0.47	0.30	0.28	0.19	0.17	0.10	0.04	0.01
			1.00	0.61	0.52	0.41	0.30	0.27	0.20	0.15	0.09	0.05	0.01
			1.01	0.66	0.55	0.43	0.23	0.25	0.18	0.16	0.08	0.05	0.01
		#2 CTCF KD mean	1.00	0.64	0.55	0.44	0.28	0.27	0.19	0.16	0.09	0.05	0.01
		#2 CTCF KD error	0.04	0.03	0.03	0.03	0.02	0.02	0.02	0.02	0.01	0.01	0.00
		#3 GFP KD raw	525	392	293	251	221	189	143	103	62	32	21
			532	400	297	259	207	186	137	90	74	40	18
			511	414	316	273	196	182	156	97	78	36	15
		#3 GFP KD norm.	1.00	0.75	0.56	0.48	0.42	0.36	0.27	0.20	0.12	0.06	0.04
			1.02	0.77	0.57	0.50	0.40	0.36	0.26	0.17	0.14	0.08	0.03
			0.98	0.79	0.60	0.52	0.38	0.35	0.30	0.19	0.15	0.07	0.03
		#3 GFP KD mean	1.00	0.77	0.58	0.50	0.40	0.36	0.28	0.18	0.14	0.07	0.03

		#3 GFP KD error	**0.04**	**0.04**	**0.03**	**0.03**	**0.03**	**0.03**	**0.02**	**0.02**	**0.02**	**0.01**	**0.01**
		#3 CTCF KD raw	**470<br>457<br>465**	**320<br>335<br>325**	**231<br>213<br>218**	**193<br>187<br>184**	**142<br>146<br>157**	**110<br>98<br>102**	**87<br>72<br>82**	**55<br>68<br>61**	**39<br>48<br>41**	**26<br>24<br>22**	**7<br>5<br>6**
		#3 CTCF KD norm.	**1.01<br>0.98<br>1.00**	**0.69<br>0.72<br>0.70**	**0.50<br>0.46<br>0.47**	**0.42<br>0.40<br>0.40**	**0.31<br>0.31<br>0.34**	**0.24<br>0.21<br>0.22**	**0.19<br>0.16<br>0.18**	**0.12<br>0.15<br>0.13**	**0.08<br>0.10<br>0.09**	**0.06<br>0.05<br>0.05**	**0.02<br>0.01<br>0.01**
		#3 CTCF KD mean<br>#3 CTCF KD error	**1.00<br>0.05**	**0.70<br>0.04**	**0.48<br>0.03**	**0.41<br>0.03**	**0.32<br>0.03**	**0.22<br>0.02**	**0.17<br>0.02**	**0.13<br>0.02**	**0.09<br>0.01**	**0.05<br>0.01**	**0.01<br>0.01**
**3C**	**U2OS**	#1 GFP KD raw	**284<br>251<br>104<br>186**	**204<br>213<br>62<br>-**	**119<br>112<br>55<br>-**	**85<br>93<br>36<br>-**	**29<br>22<br>25<br>-**	**17<br>13<br>11<br>18**	**10<br>7<br>6<br>9**	**3<br>4<br>2<br>4**	**1<br>3<br>2<br>1**	**0<br>0<br>1<br>1**	**0<br>0<br>0<br>0**
		#1 GFP KD norm.	**1.38<br>1.22<br>0.50<br>0.90**	**0.99<br>1.03<br>0.30<br>-**	**0.58<br>0.54<br>0.27<br>-**	**0.41<br>0.45<br>0.17<br>-**	**0.14<br>0.11<br>0.12<br>-**	**0.08<br>0.06<br>0.05<br>0.09**	**0.05<br>0.03<br>0.03<br>0.04**	**0.01<br>0.02<br>0.01<br>0.02**	**0.00<br>0.01<br>0.01<br>0.00**	**0.00<br>0.00<br>0.00<br>0.00**	**0.00<br>0.00<br>0.00<br>0.00**
		#1 GFP KD mean<br>#1 GFP KD error	**1.00<br>0.07**	**0.77<br>0.06**	**0.46<br>0.05**	**0.35<br>0.04**	**0.12<br>0.02**	**0.07<br>0.02**	**0.04<br>0.01**	**0.02<br>0.01**	**0.01<br>0.01**	**0.00<br>0.00**	**0.00<br>-**
		#1 CTCF KD raw	**230<br>280<br>128<br>279**	**163<br>178<br>66<br>-**	**114<br>110<br>86<br>-**	**74<br>67<br>64<br>-**	**33<br>22<br>26<br>-**	**10<br>9<br>12<br>9**	**3<br>8<br>6<br>7**	**2<br>1<br>2<br>0**	**1<br>0<br>2<br>0**	**0<br>0<br>0<br>1**	**0<br>0<br>0<br>0**
		#1 CTCF KD norm.	**1.00<br>1.22<br>0.56<br>1.22**	**0.71<br>0.78<br>0.29<br>-**	**0.50<br>0.48<br>0.38<br>-**	**0.32<br>0.29<br>0.28<br>-**	**0.14<br>0.10<br>0.11<br>-**	**0.04<br>0.04<br>0.05<br>0.04**	**0.01<br>0.03<br>0.03<br>0.03**	**0.01<br>0.00<br>0.01<br>0.00**	**0.00<br>0.00<br>0.01<br>0.00**	**0.00<br>0.00<br>0.00<br>0.00**	**0.00<br>0.00<br>0.00<br>0.00**
		#1 CTCF KD mean<br>#1 CTCF KD error	**1.00<br>0.07**	**0.59<br>0.05**	**0.45<br>0.04**	**0.30<br>0.04**	**0.12<br>0.02**	**0.04<br>0.01**	**0.03<br>0.01**	**0.01<br>0.00**	**0.00<br>0.00**	**0.00<br>0.00**	**0.00<br>-**
		#2 GFP KD raw	**345<br>350<br>362**	**293<br>301<br>304**	**170<br>187<br>190**	**139<br>141<br>145**	**46<br>40<br>49**	**19<br>21<br>26**	**7<br>9<br>11**	**5<br>3<br>5**	**3<br>1<br>2**	**1<br>1<br>0**	**0<br>0<br>0**
		#2 GFP KD norm.	**0.98<br>0.99<br>1.03**	**0.83<br>0.85<br>0.86**	**0.48<br>0.53<br>0.54**	**0.39<br>0.40<br>0.41**	**0.13<br>0.11<br>0.14**	**0.05<br>0.06<br>0.07**	**0.02<br>0.03<br>0.03**	**0.01<br>0.01<br>0.01**	**0.01<br>0.00<br>0.01**	**0.00<br>0.00<br>0.00**	**0.00<br>0.00<br>0.00**
		#2 GFP KD mean<br>#2 GFP KD error	**1.00<br>0.05**	**0.85<br>0.05**	**0.52<br>0.04**	**0.40<br>0.03**	**0.13<br>0.02**	**0.06<br>0.01**	**0.03<br>0.01**	**0.01<br>0.01**	**0.01<br>0.00**	**0.00<br>0.00**	**0.00<br>-**
		#2 CTCF KD raw	**300<br>297<br>333**	**214<br>210<br>206**	**133<br>114<br>125**	**88<br>93<br>95**	**29<br>24<br>31**	**13<br>8<br>9**	**4<br>3<br>5**	**0<br>1<br>2**	**1<br>1<br>0**	**0<br>0<br>0**	**0<br>0<br>0**
		#2 CTCF KD norm.	**0.97<br>0.96<br>1.07**	**0.69<br>0.68<br>0.66**	**0.43<br>0.37<br>0.40**	**0.28<br>0.30<br>0.31**	**0.09<br>0.08<br>0.10**	**0.04<br>0.03<br>0.03**	**0.01<br>0.01<br>0.02**	**0.00<br>0.00<br>0.01**	**0.00<br>0.00<br>0.00**	**0.00<br>0.00<br>0.00**	**0.00<br>0.00<br>0.00**
		#2 CTCF KD mean<br>#2 CTCF KD error	**1.00<br>0.06**	**0.68<br>0.05**	**0.40<br>0.04**	**0.30<br>0.03**	**0.09<br>0.02**	**0.03<br>0.01**	**0.01<br>0.01**	**0.00<br>0.00**	**0.00<br>0.00**	**0.00<br>-**	**0.00<br>-**

		#3 GFP $^{\mathrm{KD\ raw}}$	**194<br>190<br>187**	**158<br>164<br>160**	**97<br>103<br>99**	**68<br>82<br>79**	**25<br>27<br>20**	**12<br>10<br>9**	**5<br>4<br>7**	**0<br>3<br>1**	**1<br>1<br>0**	**0<br>0<br>0**	**0<br>0<br>0**
		#3 GFP $^{\mathrm{KD\ norm.}}$	**1.02<br>1.00<br>0.98**	**0.83<br>0.86<br>0.84**	**0.51<br>0.54<br>0.52**	**0.36<br>0.43<br>0.42**	**0.13<br>0.14<br>0.11**	**0.06<br>0.05<br>0.05**	**0.03<br>0.02<br>0.04**	**0.00<br>0.02<br>0.01**	**0.01<br>0.01<br>0.00**	**0.00<br>0.00<br>0.00**	**0.00<br>0.00<br>0.00**
		#3 GFP $^{\mathrm{KD\ mean}}$<br>#3 GFP $^{\mathrm{KD\ error}}$	**1.00<br>0.07**	**0.84<br>0.07**	**0.52<br>0.05**	**0.40<br>0.05**	**0.13<br>0.03**	**0.05<br>0.02**	**0.03<br>0.01**	**0.01<br>0.01**	**0.00<br>0.00**	**0.00<br>-**	**0.00<br>-**
		#3 CTCF $^{\mathrm{KD\ raw}}$	**139<br>141<br>130**	**84<br>85<br>79**	**60<br>57<br>53**	**39<br>35<br>40**	**13<br>12<br>15**	**5<br>4<br>6**	**2<br>3<br>2**	**1<br>0<br>1**	**0<br>0<br>0**	**0<br>0<br>0**	**0<br>0<br>0**
		#3 CTCF $^{\mathrm{KD\ norm.}}$	**1.02<br>1.03<br>0.95**	**0.61<br>0.62<br>0.58**	**0.44<br>0.42<br>0.39**	**0.29<br>0.26<br>0.29**	**0.10<br>0.09<br>0.11**	**0.04<br>0.03<br>0.04**	**0.01<br>0.02<br>0.01**	**0.01<br>0.00<br>0.01**	**0.00<br>0.00<br>0.00**	**0.00<br>0.00<br>0.00**	**0.00<br>0.00<br>0.00**
		#3 CTCF $^{\mathrm{KD\ mean}}$<br>#3 CTCF $^{\mathrm{KD\ error}}$	**1.00<br>0.09**	**0.60<br>0.07**	**0.41<br>0.06**	**0.28<br>0.05**	**0.10<br>0.03**	**0.04<br>0.02**	**0.02<br>0.01**	**0.00<br>0.01**	**0.00<br>-**	**0.00<br>-**	**0.00<br>-**
8	**AID**	#1 – 0 µM $^{\mathrm{AUX\ raw}}$<br>#1 – 0 µM $^{\mathrm{AUX\ norm.}}$	**309<br>1.00**	**263<br>0.85**	**226<br>0.73**	**170<br>0.55**	**154<br>0.50**	**142<br>0.46**	**118<br>0.38**	**85<br>0.28**	**73<br>0.24**	**52<br>0.17**	**24<br>0.08**
		#1 – 25 µM $^{\mathrm{AUX\ raw}}$<br>#1 – 25 µM $^{\mathrm{norm.}}$	**263<br>1.00**	**210<br>0.80**	**176<br>0.67**	**150<br>0.57**	**141<br>0.54**	**65<br>0.25**	**53<br>0.20**	**42<br>0.16**	**33<br>0.13**	**25<br>0.10**	**15<br>0.06**
		#1 – 500 µM $^{\mathrm{AUX\ raw}}$<br>#1 – 500 µM $^{\mathrm{AUX\ norm.}}$	**280<br>1.00**	**223<br>0.80**	**153<br>0.55**	**90<br>0.32**	**82<br>0.29**	**74<br>0.26**	**66<br>0.24**	**53<br>0.19**	**31<br>0.11**	**24<br>0.09**	**17<br>0.06**
		#1 – 1000 µM $^{\mathrm{AUX\ raw}}$<br>#1 – 1000 µM $^{\mathrm{AUX\ norm.}}$	**268<br>1.00**	**206<br>0.77**	**165<br>0.62**	**104<br>0.39**	**92<br>0.34**	**85<br>0.32**	**76<br>0.28**	**54<br>0.20**	**43<br>0.16**	**26<br>0.10**	**13<br>0.05**
		#2 – 0 µM $^{\mathrm{AUX\ raw}}$<br>#2 – 0 µM $^{\mathrm{AUX\ norm.}}$	**473<br>1.00**	**418<br>0.88**	**387<br>0.82**	**328<br>0.69**	**261<br>0.55**	**188<br>0.40**	**159<br>0.34**	**101<br>0.21**	**89<br>0.19**	**60<br>0.13**	**43<br>0.09**
		#2 – 25 µM $^{\mathrm{AUX\ raw}}$<br>#2 – 25 µM $^{\mathrm{norm.}}$	**430<br>1.00**	**375<br>0.87**	**353<br>0.82**	**270<br>0.63**	**212<br>0.49**	**184<br>0.43**	**137<br>0.32**	**110<br>0.26**	**69<br>0.16**	**56<br>0.13**	**30<br>0.07**
		#2 – 500 µM $^{\mathrm{AUX\ raw}}$<br>#2 – 500 µM $^{\mathrm{AUX\ norm.}}$	**433<br>1.00**	**383<br>0.88**	**324<br>0.75**	**279<br>0.64**	**214<br>0.49**	**171<br>0.39**	**115<br>0.27**	**101<br>0.23**	**74<br>0.17**	**67<br>0.15**	**20<br>0.05**
		#2 – 1000 µM $^{\mathrm{AUX\ raw}}$<br>#2 – 1000 µM $^{\mathrm{AUX\ norm.}}$	**430<br>1.00**	**358<br>0.83**	**323<br>0.75**	**257<br>0.60**	**234<br>0.54**	**150<br>0.35**	**130<br>0.30**	**95<br>0.22**	**55<br>0.13**	**42<br>0.10**	**20<br>0.05**
		#3 – 0 µM $^{\mathrm{AUX\ raw}}$<br>#3 – 0 µM $^{\mathrm{AUX\ norm.}}$	**430<br>1.00**	**370<br>0.86**	**340<br>0.79**	**270<br>0.63**	**240<br>0.56**	**153<br>0.36**	**124<br>0.29**	**97<br>0.23**	**76<br>0.18**	**57<br>0.13**	**36<br>0.08**
		#3 – 25 µM $^{\mathrm{AUX\ raw}}$<br>#3 – 25 µM $^{\mathrm{norm.}}$	**376<br>1.00**	**336<br>0.89**	**275<br>0.73**	**205<br>0.55**	**180<br>0.48**	**134<br>0.36**	**110<br>0.29**	**93<br>0.25**	**64<br>0.17**	**53<br>0.14**	**25<br>0.07**
		#3 – 500 µM $^{\mathrm{AUX\ raw}}$<br>#3 – 500 µM $^{\mathrm{AUX\ norm.}}$	**427<br>1.00**	**368<br>0.86**	**327<br>0.77**	**230<br>0.54**	**200<br>0.47**	**170<br>0.40**	**128<br>0.30**	**100<br>0.23**	**73<br>0.17**	**59<br>0.14**	**16<br>0.04**
		#3 – 1000 µM $^{\mathrm{AUX\ raw}}$<br>#3 – 1000 µM $^{\mathrm{AUX\ norm.}}$	**400<br>1.00**	**345<br>0.86**	**286<br>0.72**	**221<br>0.55**	**158<br>0.40**	**121<br>0.30**	**109<br>0.27**	**79<br>0.20**	**54<br>0.14**	**31<br>0.08**	**17<br>0.04**
		#4 – 0 µM $^{\mathrm{AUX\ raw}}$<br>#4 – 0 µM $^{\mathrm{AUX\ norm.}}$	**399<br>1.00**	**354<br>0.89**	**324<br>0.81**	**270<br>0.68**	**194<br>0.49**	**160<br>0.40**	**121<br>0.30**	**91<br>0.23**	**77<br>0.19**	**44<br>0.11**	**37<br>0.09**

		#4 – 25 μM $^{\text{AUX raw}}$<br>#4 – 25 μM $^{\text{norm.}}$	**284**<br>**1.00**	**249**<br>**0.88**	**233**<br>**0.82**	**175**<br>**0.62**	**135**<br>**0.48**	**118**<br>**0.42**	**86**<br>**0.30**	**66**<br>**0.23**	**47**<br>**0.17**	**32**<br>**0.11**	**18**<br>**0.06**
		#4 – 500 μM $^{\text{AUX raw}}$<br>#4 – 500 μM $^{\text{AUX norm.}}$	**346**<br>**1.00**	**304**<br>**0.88**	**263**<br>**0.76**	**217**<br>**0.63**	**162**<br>**0.47**	**113**<br>**0.33**	**84**<br>**0.24**	**52**<br>**0.15**	**39**<br>**0.11**	**30**<br>**0.09**	**17**<br>**0.05**
		#4 – 1000 μM $^{\text{AUX raw}}$<br>#4 – 1000 μM $^{\text{AUX norm.}}$	**338**<br>**1.00**	**230**<br>**0.68**	**210**<br>**0.62**	**166**<br>**0.49**	**117**<br>**0.35**	**101**<br>**0.30**	**84**<br>**0.25**	**52**<br>**0.15**	**30**<br>**0.09**	**21**<br>**0.06**	**19**<br>**0.06**
		0 μM $^{\text{AUX mean}}$<br>0 μM $^{\text{AUX error}}$	**1.00**<br>**0.05**	**0.87**<br>**0.05**	**0.79**<br>**0.04**	**0.64**<br>**0.04**	**0.52**<br>**0.04**	**0.40**<br>**0.03**	**0.33**<br>**0.03**	**0.24**<br>**0.02**	**0.20**<br>**0.02**	**0.13**<br>**0.02**	**0.09**<br>**0.01**
		25 μM $^{\text{AUX mean}}$<br>25 μM ^{error}	**1.00**<br>**0.05**	**0.86**<br>**0.05**	**0.76**<br>**0.05**	**0.59**<br>**0.04**	**0.50**<br>**0.04**	**0.36**<br>**0.03**	**0.28**<br>**0.03**	**0.22**<br>**0.03**	**0.16**<br>**0.02**	**0.12**<br>**0.02**	**0.06**<br>**0.01**
		500 μM $^{\text{AUX mean}}$<br>500 μM $^{\text{AUX error}}$	**1.00**<br>**0.05**	**0.86**<br>**0.05**	**0.71**<br>**0.04**	**0.53**<br>**0.04**	**0.43**<br>**0.03**	**0.35**<br>**0.03**	**0.26**<br>**0.03**	**0.20**<br>**0.02**	**0.14**<br>**0.02**	**0.12**<br>**0.02**	**0.05**<br>**0.01**
		1000 μM $^{\text{AUX mean}}$<br>1000 μM $^{\text{AUX error}}$	**1.00**<br>**0.05**	**0.79**<br>**0.05**	**0.68**<br>**0.04**	**0.51**<br>**0.04**	**0.41**<br>**0.03**	**0.32**<br>**0.03**	**0.28**<br>**0.03**	**0.19**<br>**0.02**	**0.13**<br>**0.02**	**0.08**<br>**0.02**	**0.05**<br>**0.01**

***raw = raw number of colonies counted; ** norm. = normalized to the average of the respective unirradiated samples; ***mean & error = mean of normalized values and calculated relative error values (see calculation below), respectively shown as dots and error bars in the survival curves;**
cell line name abbreviations: HK = HeLa Kyoto, AID = mESC-AID-CTCF;
sample names: # = biological replicate number, AUX = auxin.

$$Relative\ error = mean\ normalized\ number\ of\ colonies\ x \frac{1}{\sqrt{(mean\ raw\ number\ of\ colonies)}}$$

Supplementary Figure S4: Colony formation images.

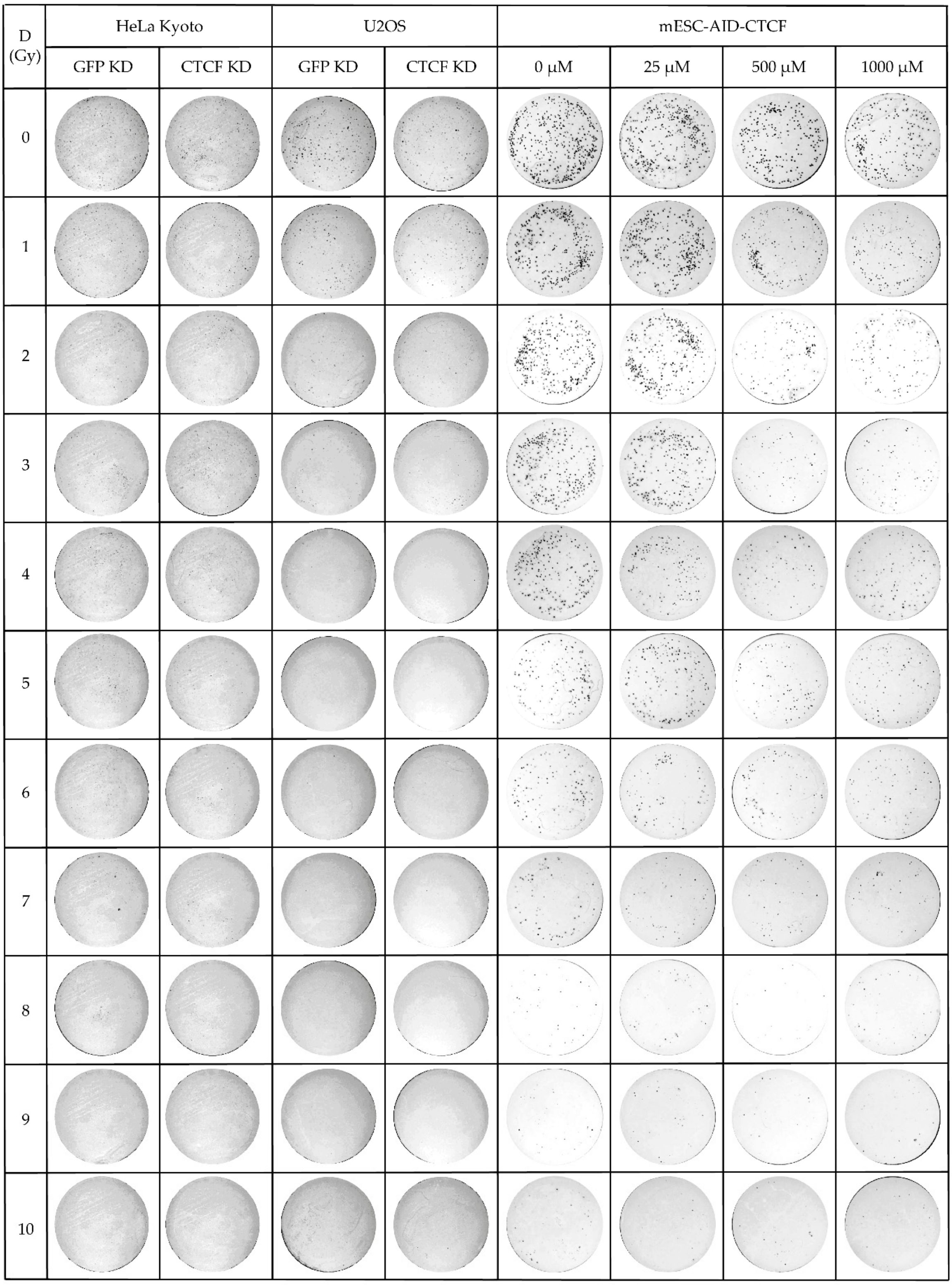

A complete set of images of colony formation of HeLa Kyoto, U2OS and mESC-AID-CTCF cells is shown (see methods section 4.6. for details). D (Gy) = X-rays dose; 0 – 1000 μM = auxin concentration.

Supplementary Figure S5. Survival curves and modeling predictions with equal y axis.

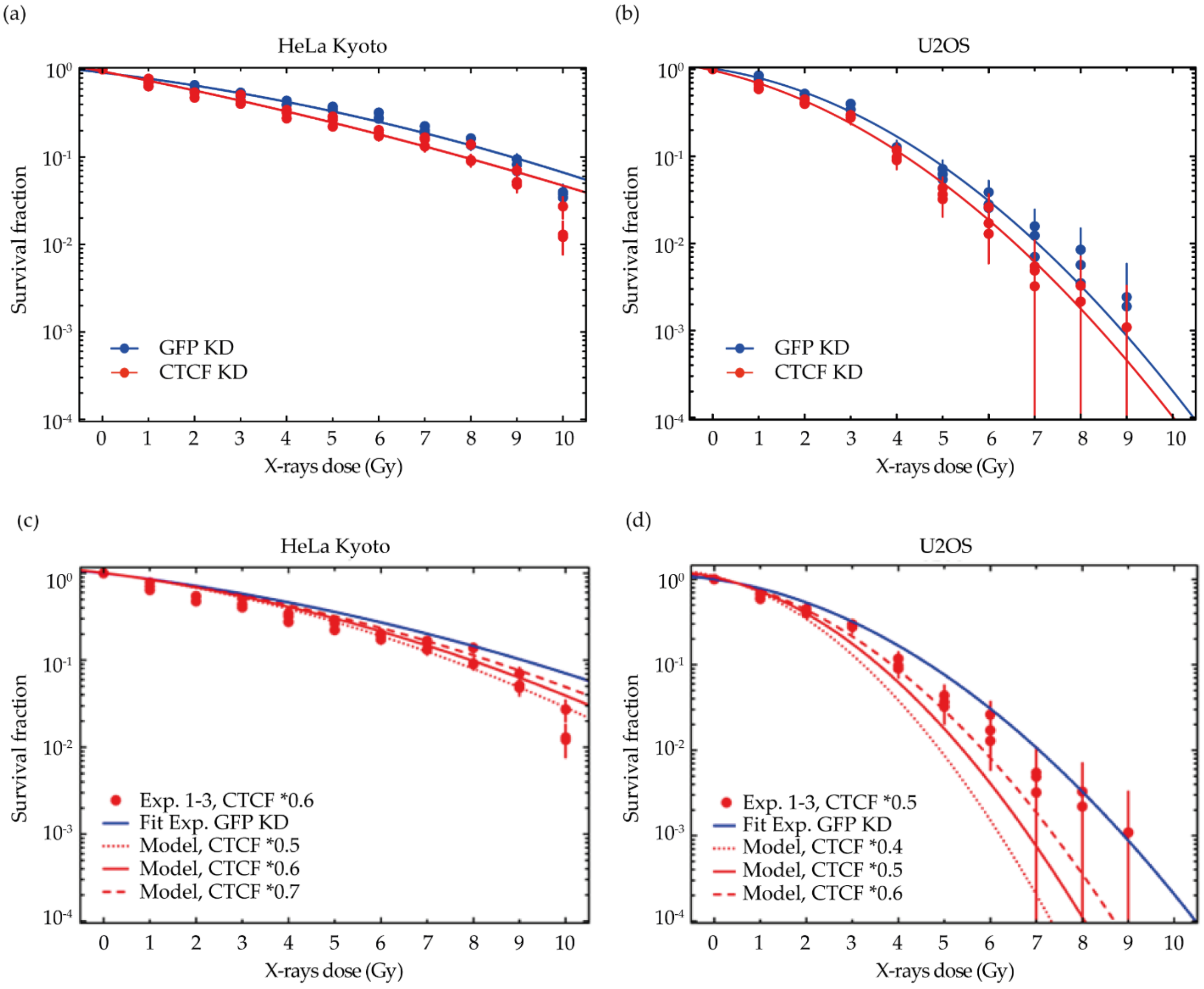

The experimental survival curves (Figure 3) and relative modeling prediction (Figure 4) of HeLa Kyoto and U2OS were plotted with a different scale on the y axis, due to the very different radiosensitivity of the two cell lines, with the scaling that better suited their visualization. Here both the experimental data (**a**, **b**) and the model predictions (**c**, **d**) are plotted by using the same scaling that was chosen for U2OS, to allow direct comparison. Whiskers = error bars (see the footer of Supplementary Table S6 for the calculation of the relative error); fitting curve weighted by $\frac{1}{relative\ error^2}$.

Supplementary Figure S6. Time course validation of the CTCF-degron kinetics.

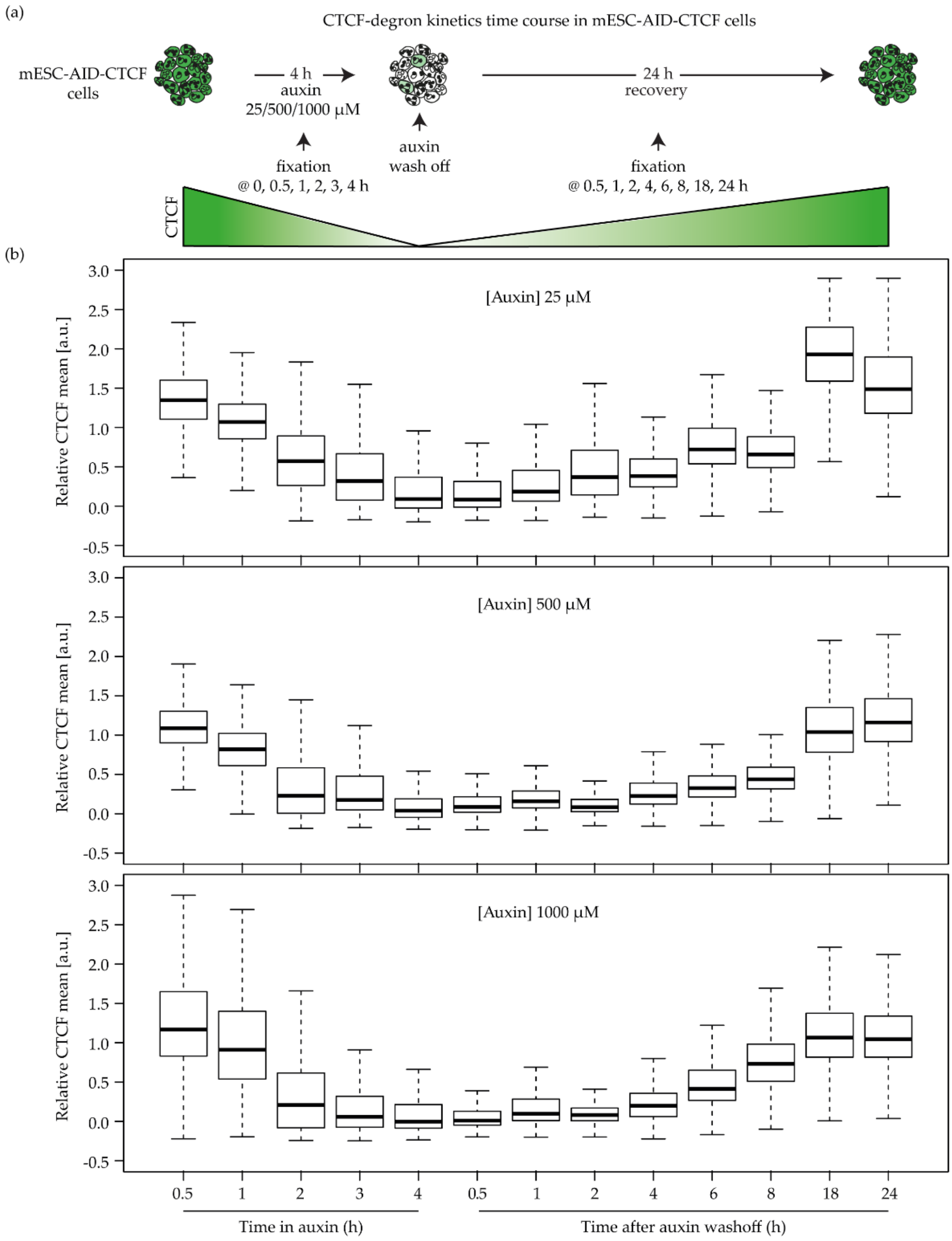

[Auxin]	Median												
25 uM	1.35	1.07	0.57	0.32	0.09	0.08	0.18	0.37	0.38	0.72	0.66	1.93	1.49
500 uM	1.09	0.82	0.23	0.17	0.04	0.09	0.16	0.08	0.23	0.33	0.44	1.04	1.16
1000 uM	1.17	0.91	0.21	0.06	-0.002	0.01	0.10	0.08	0.20	0.41	0.73	1.07	1.04

(**a**) Experimental scheme of the time course experiment: different auxin concentrations (0, 25, 500, 1000 μM) were applied for 4 hours to mESC-AID-CTCF cells, then auxin was washed off and replaced with fresh medium to allow CTCF recovery; cells were collected at different time points during the auxin treatment and upon washing off, imaged with high content microscopy (Supplementary Table S3) and CTCF intensity values were measured (Supplementary Figure SF); (**b**) After subtracting the background measured in untagged ES-E14TG2a WT, the mean CTCF-GFP intensities were normalized to (divided by) the median value of the respective time 0 h of each auxin treatment and plotted as boxplots (see Supplementary Figure S3 for boxplot interpretation). The results are based on three biological replicates.

Supplementary Figure S7: Screenshot of model implementation as Excel sheet

	A	B
1	**Input Parameters**	
2	**6000**	**Genome Size [Mbp]**
3	**2,0**	**Domain Size, Ctrl [Mbp]**
4	**0,200**	**alpha_ref [1/Gy]**
5	**0,020**	**beta_ref [1/Gy^2]**
6	**0,500**	**CTCF Depletion Factor**
7	**Derived Parameters, general**	
8	500	Nuclear Volume [um3]
9	30	Haploid YDSB [1/Gy]
10	6,6667E-03	ε_i
11	1,4667E-01	ε_c
12	**Derived Parameters, Ctrl specific**	
13	0,550	Ldomain, Ctrl [um]
14	0,167	Vdomain, Ctrl [um3]
15	3000	Ndomain, Ctrl
16	**Derived Parameters, CTCF specific**	
17	2,000	1/CTCF-Fact.
18	0,693	LDomain, CTCF KD [um]
19	0,333	Vdomain, CTCF KD [um3]
20	1500	NDomain, CTCF KD
21	4,0	Domain Size, CTCF KD [Mbp]

Blatt anzeigen oder ausblenden.

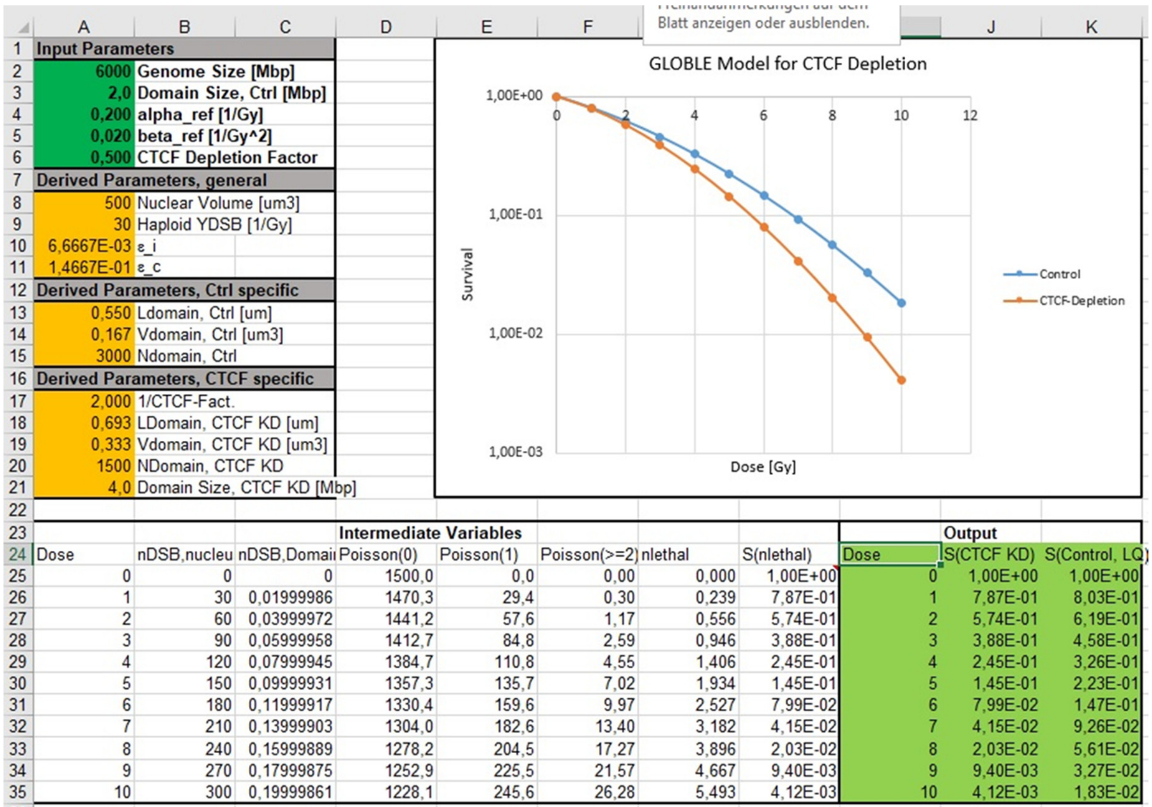

	A	B	C	D	E	F	G	H	I	J	K
23				**Intermediate Variables**						**Output**	
24	Dose	nDSB,nucleu	nDSB,Domai	Poisson(0)	Poisson(1)	Poisson(>=2)	nlethal	S(nlethal)	Dose	S(CTCF KD)	S(Control, LQ)
25	0	0	0	1500,0	0,0	0,00	0,000	1,00E+00	0	1,00E+00	1,00E+00
26	1	30	0,01999986	1470,3	29,4	0,30	0,239	7,87E-01	1	7,87E-01	8,03E-01
27	2	60	0,03999972	1441,2	57,6	1,17	0,556	5,74E-01	2	5,74E-01	6,19E-01
28	3	90	0,05999958	1412,7	84,8	2,59	0,946	3,88E-01	3	3,88E-01	4,58E-01
29	4	120	0,07999945	1384,7	110,8	4,55	1,406	2,45E-01	4	2,45E-01	3,26E-01
30	5	150	0,09999931	1357,3	135,7	7,02	1,934	1,45E-01	5	1,45E-01	2,23E-01
31	6	180	0,11999917	1330,4	159,6	9,97	2,527	7,99E-02	6	7,99E-02	1,47E-01
32	7	210	0,13999903	1304,0	182,6	13,40	3,182	4,15E-02	7	4,15E-02	9,26E-02
33	8	240	0,15999889	1278,2	204,5	17,27	3,896	2,03E-02	8	2,03E-02	5,61E-02
34	9	270	0,17999875	1252,9	225,5	21,57	4,667	9,40E-03	9	9,40E-03	3,27E-02
35	10	300	0,19999861	1228,1	245,6	26,28	5,493	4,12E-03	10	4,12E-03	1,83E-02

A simple implementation of the model as Excel sheet is deposited in the Supplementary Materials (see section 4.7).

References

1. Erfle, H.; Neumann, B.; Liebel, U.; Rogers, P.; Held, M.; Walter, T.; Ellenberg, J.; Pepperkok, R. Reverse transfection on cell arrays for high content screening microscopy. *Nat. Protoc.* **2007**, *2*, 392–399, doi:10.1038/nprot.2006.483.
2. Pontén, J.; Saksela, E. Two established in vitro cell lines from human mesenchymal tumours. *Int. J. Cancer* **1967**, *2*, 434–447.
3. Nora, E.P.; Goloborodko, A.; Valton, A.-L.; Gibcus, J.H.; Uebersohn, A.; Abdennur, N.; Dekker, J.; Mirny, L.A.; Bruneau, B.G. Targeted Degradation of CTCF Decouples Local Insulation of Chromosome Domains from Genomic Compartmentalization. *Cell* **2017**, *169*, 930-944.e22, doi:10.1016/j.cell.2017.05.004.
4. Hooper, M.; Hardy, K.; Handyside, A.; Hunter, S.; Monk, M. HPRT-deficient (Lesch-Nyhan) mouse embryos derived from germline colonization by cultured cells. *Nature* **1987**, *326*, 292–295, doi:10.1038/326292a0.
